# Machine Learning Empowered a Graphical User Interface on Native Fluorescence to Predict Breast Cancer

**DOI:** 10.1021/acsomega.4c11669

**Published:** 2025-05-14

**Authors:** Ashwini Amin, Mallika Priya, Jackson Rodrigues, Shimul Biswas, Subhash Chandra, Stanley Mathew, Satadru Ray, Bola Sadashiva Satish Rao, Krishna Kishore Mahato

**Affiliations:** † Department of Biophysics, Manipal School of Life Sciences, 539679 Manipal Academy of Higher Education, Manipal 576104, Karnataka, India; ‡ Department of Computer Science & Engineering, Manipal Institute of Technology, Manipal Academy of Higher Education, Manipal 576104, Karnataka, India; § Clinical Mind, Boston, Massachusetts 02108, United States; ∥ Department of General Surgery, Kasturba Medical College, Manipal Academy of Higher Education, Manipal 576104, Karnataka, India; ⊥ Department of General Surgery, Kasturba Medical College, Manipal Academy of Higher Education, Mangaluru 575001, Karnataka, India; # Department of Radiation Biology & Toxicology, Manipal School of Life Sciences, Manipal Academy of Higher Education, Manipal 576104, Karnataka, India

## Abstract

Breast cancer poses a significant global health challenge, requiring improved diagnostic solutions for its timely intervention and treatment. Real-time diagnostic approaches in current practice offer promising avenues for early detection. However, these techniques often lack specificity, necessitating the development of robust diagnostic tools for real-time applications. In the current study, fluorescence spectroscopy is integrated with machine learning algorithms, and a graphical user interface (GUI) is developed for rapid breast cancer prediction. This study records 206 native fluorescence spectra, 103 spectra each from 31 normal and 31 malignant breast tissues using 325 nm excitation, followed by discrimination analysis using different machine learning algorithms, including backpropagation artificial neural network (BP-ANN), support vector machine (SVM), and Naïve Bayes (NB). Comparative analysis reveals that SVM in combination with a polynomial kernel demonstrated the superior performance of accuracy (98.78%), sensitivity (100%), specificity (97.56%), and precision (97.62%), among others. Furthermore, the in-house developed GUI applied to the current data showed the possibility of real-time prediction of pathological breast tissues, facilitating standalone applications.

## Graphical abstract

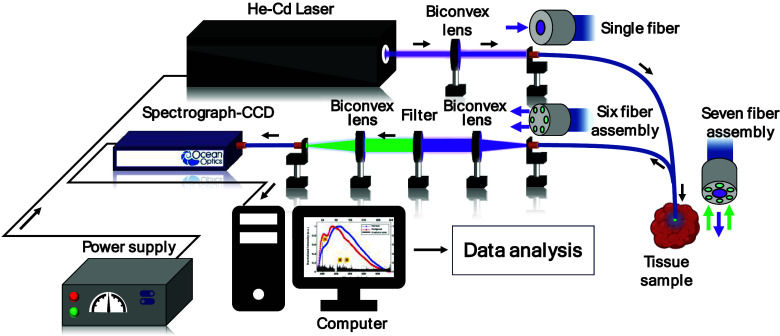

## 1 Introduction

Despite advancements in medical sciences and technology, cancer remains a significant global health concern. According to The Global Cancer Observatory, female breast cancer ranked number one among the rest of the cancers.^1^ Traditional screening methods, such as mammography, while effective for early detection and treatments, often yield false-positive results in the case of dense breasts.^2^ Therefore, there is an urgent need for the development of efficient, fast, accurate, and rapid screening technologies for the early detection and diagnosis of breast cancer. Optical spectroscopy, including native fluorescence/autofluorescence, has shown immense potential to study cellular components and tissue pathology.^3–6^ In autofluorescence, the emission is due to an intrinsic fluorophore present in the specimen under study. It is a phenomenon that appears when intrinsic fluorophores get excited at their suitable excitation wavelengths. Several intrinsic fluorophores in the biological specimens show fluorescence properties.^7,8^ Alterations in the tissue structure, metabolic activity, and the tumor microenvironment related to breast cancer can change the concentration and distribution of fluorophores such as collagen, elastin, nicotinamide adenine dinucleotide hydride (NADH), flavin adenine dinucleotide (FAD), etc., resulting in observable differences in their absorbance and fluorescence spectra.^9,10^ Autofluorescence spectroscopy, which exploits the natural fluorescence properties of endogenous fluorophores in cells and tissues at appropriate excitations, has shown high sensitivity with reduced sample preparation steps, making it suitable for studying various cancer types.^11–15^ Several research studies have demonstrated the efficacy of fluorescence spectroscopy in cancer diagnosis using machine learning algorithms, where Stokes shift spectroscopy,^16^ synchronous luminescence spectroscopy,^17^ and native fluorescence spectroscopy^18^ are discussed.

The application of machine learning algorithms to spectral data analysis for differentiation and discrimination of various tissue pathological conditions has revolutionized the field of biomedical spectroscopy. Researchers around the globe have shown several-fold improvement in the classification of spectral data by extracting meaningful information from them using various statistical tools, including machine learning^19,20^ such as linear discriminant analysis (LDA),^21^ principal component analysis (PCA),^22^ support vector machine (SVM),^23,24^ and many more.^25–29^ These algorithms analyze spectral data to identify key features that differentiate between normal and malignant tissues, leading to accurate classification and diagnosis of cancer. While techniques such as PCA have been widely used for feature reduction, they may suffer from limitations such as information loss and susceptibility to noise.^30^ Researchers advocate for feature selection techniques over feature extraction methods to address these challenges. Feature selection helps eliminate noise and redundant features, improving the accuracy and reliability of cancer diagnosis.^31^

Among various feature selection methods, Minimum Redundancy Maximum Relevance (mRMR) offers distinct advantages by simultaneously maximizing feature relevance to the target class while minimizing redundancy among selected features, making it particularly effective for spectral data where adjacent wavelengths exhibit high correlation.^32^ Unlike transformation methods such as PCA, mRMR preserves the original features rather than creating abstract components, maintaining the physical interpretability of spectral bands for more meaningful analysis.^33^ Furthermore, mRMR demonstrates superior computational efficiency compared to wrapper methods while handling feature interactions more effectively than univariate approaches, resulting in more robust models that are less prone to overfitting when analyzing high-dimensional spectral data sets.^34,35^

Fluorescence spectroscopy, combined with advanced data analysis techniques, holds great promise for the early detection and diagnosis of breast cancer.^36,37^ By leveraging machine learning algorithms and innovative spectroscopy methods, it is possible to achieve high accuracy and reliability in cancer classification, leading to better patient outcomes and reducing the burden of this devastating disease on society. Further research and development in this field are essential to realizing the full potential of optical spectroscopy in cancer diagnosis and treatment. Therefore, in the present study, native fluorescence spectra of normal and breast tumor tissues were recorded at 325 nm excitation and subjected to machine learning-based analysis for discrimination using proper training and testing models. Further, an attempt has been made to develop a GUI-based machine learning analysis to predict unknown new data for standalone applications.

## 2 Materials and Methods

### 2.1 Experimental Setup

Figure 1 shows the experimental setup used in the current study. The setup consists of a He–Cd laser (325 nm) as an excitation source, a 7-fiber-based optical probe (1 for excitation and 6 for collection of native fluorescence), and an Ocean Optics-QE Pro spectrograph (grating: 300 lines/mm, blazed at 300 nm) for spectral dispersion and detection. The setup also consists of a longpass filter (365 nm longpass filter, Schneider Kreuznach, Germany) to avoid Rayleigh scattered light and a computer for spectral recording and storing.

**Figure 1:**
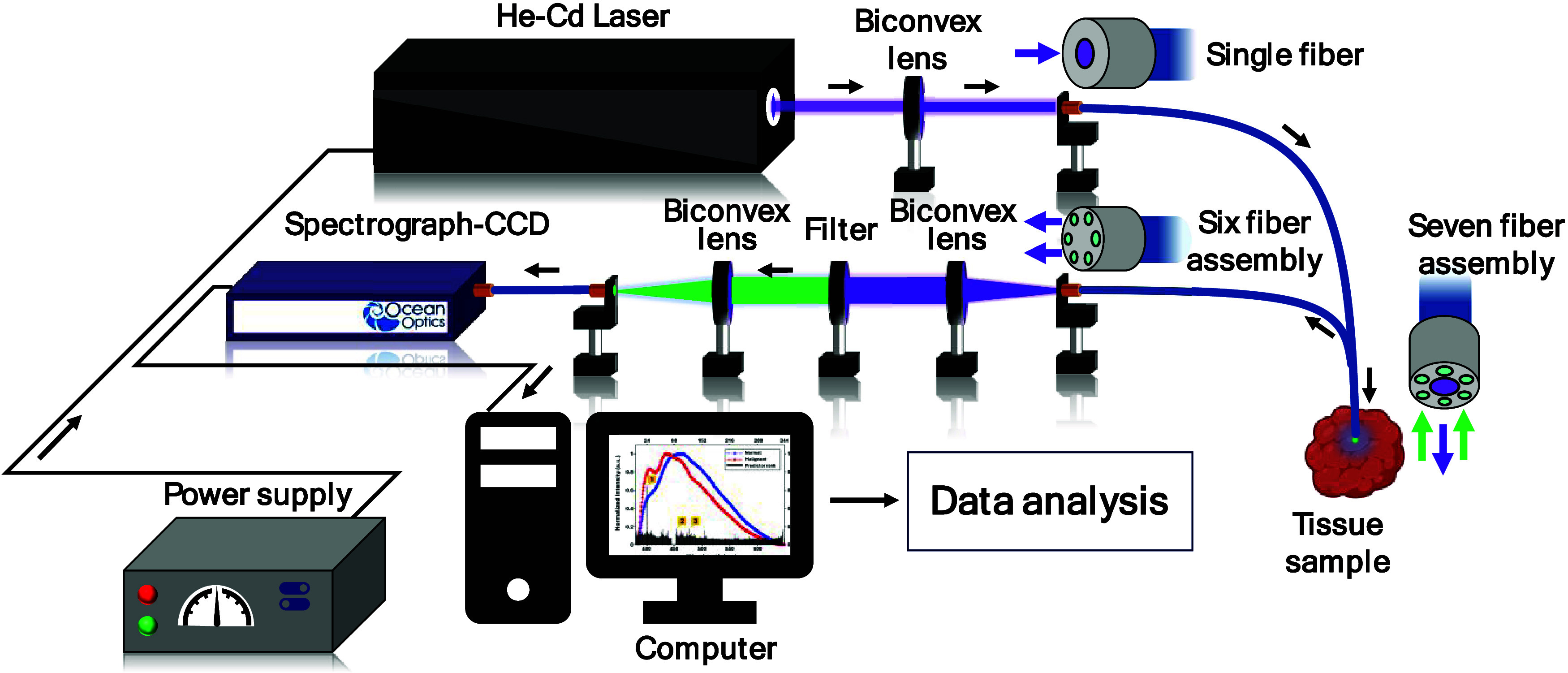
Instrumentation setup used to record native fluorescence spectra of normal and malignant breast tissues *ex vivo* at 325 nm excitation. Various components of the setup are marked as He–Cd laser, power supply, optical lenses, YZ translational stage with a fiber coupler and an SMA adapter, fiber probe consisting of a single fiber, seven fiber assembly, six fiber assembly (200 μm) for collection of native fluorescence, longpass filter (allowing >365 nm), spectrograph-CCD for spectral dispersion and detection, and a computer for recording/storing native fluorescence data.

### 2.2 Data Collection and Handling

After obtaining the institutional ethics committee approval, surgically resected malignant breast tissue samples were collected from the consenting patients. The control samples were selected from the uninvolved areas of the same samples under study. The collected breast tissues were of different sizes, ranging from approximately 15 to 20 mm^2^ in surface area and a thickness of 2–4 mm. The samples were mounted on a quartz plate and excited by a 325 nm He–Cd laser for recording the corresponding native fluorescence spectra using a spectrograph in the spectral range of 375–650 nm. The samples were kept moist with normal saline during spectral acquisition, and three spectra were recorded at 3–4 different sites on every sample. Three spectra in each location were averaged to obtain the mean spectrum, which was used for discrimination analysis of the malignant samples from the normal. Thus, a total of 206 spectra (103 normal, 103 malignant) from 31 normal and 31 malignant tissue samples from 31 different patients were obtained in this study. The cancer tissues were collected from 31 patients, with the majority classified as grade II or grade III and at stages II to III.

### 2.3 Data Analysis

#### 2.3.1 Preprocessing of the Spectra

The recorded spectra in the present study were subjected to baseline correction, region of interest selection, smoothing using filter functions to remove unwanted noise signals, and, finally, normalization with respect to the highest peak in the spectra. This was carried out using in-house built code implemented using MATLAB v.2019b.

#### 2.3.2 Feature Selection

In the current study, a filter-based feature selection algorithm known as Minimum Redundancy Maximum Relevance (mRMR) was used. The mRMR was selected for its ability to identify features that are highly relevant to the classification task while minimizing redundancy among the selected features, which is particularly important in spectral data where adjacent wavelengths may be highly correlated. To minimize the correlation between different features of a target class and maximize the correlation within the class, the mRMR uses mutual information. After applying this method, the feature ranking was obtained based on its importance,^38^ and the top 3 features at wavelengths 400.6 nm (feature 1), 453.2 nm (feature 2), and 477.6 nm (feature 3) were selected for further analysis.

#### 2.3.3 Spectral Intensity Ratio Calculation on Selected Features

The intensity ratios of selected features 2 and 3 versus feature 1 for each of the normalized spectra under study were calculated, as shown in Table 1. These ratios were subsequently used as the input feature matrix for the machine learning model for training and testing.

**Table 1:** Details of the Intensity Ratios of Features in Normalized Spectra

normalized intensity ratios	intensity corresponding to	features corresponding wavelengths (nm)
R1	feature 2/feature 1	453.2/400.6
R2	feature 3/feature 1	477.6/400.6

### 2.4 Machine Learning

In the current study, a comparison of the performances of artificial neural networks (ANNs) was made with different training algorithms like resilient backpropagation (RP), scaled conjugate gradient (SCG), and Gaussian discriminant analysis (GDA). Similarly, support vector machine (SVM) learning algorithms with different kernel functions, radial basis function (RBF), polynomial, and linear. Likewise, Naïve Bayes-based classification analysis was also attempted in the study. To ensure the generalizability of the model, we employed *k*-fold cross-validation (*k* = 5) during the training process.

### 2.5 Graphical User Interface Using MATLAB

In the current study, a MATLAB-based GUI was designed and developed to predict new spectral data that can be used by anybody without prior coding knowledge. In actual practice, user interfaces wait for the response from the end-user to perform any operation, like “clicking on a button” and provide the performance outcomes in the form of a graph or text message, etc. By building these interfaces, anyone without knowledge of the development of the code can use it easily for the analysis of the data. Also, the GUI can be installed in any system without the knowledge of MATLAB coding for standalone applications, thereby making it suitable for user-friendly and remote applications.^39^ It is an interactive platform for real-time classification of data, enabling efficient differentiation between two or more classes of data. It simplifies data analysis by integrating the trained machine learning model at the back end, making it accessible for clinical and research applications. Currently, the GUI is the most prevalent and recognized user interface for computers. It has an input and an output component. The measured data, upon spectral preprocessing or selected spectral features from the data of a study, are fed to the input of the GUI in a particular format, which upon specific operation provides the required outputs in a particular format. The input and output information on the GUI is not specific to any particular type of spectral pattern/data, but can be extended to any other type of data. The GUI designed in this study accepts spectral data files in *.spc* format, displaying the corresponding 2D fluorescence spectrum alongside presaved reference spectra for normal and malignant breast tissue. Upon loading the spectral data, the file name appears in the status bar, ensuring traceability. The system applies preprocessing steps, including spectral normalization, to enhance data consistency before classification. When the “Predict” button is pressed, the model classifies the spectrum, triggering a green indicator for normal tissue or a red indicator for malignant tissue. Additionally, users can generate a diagnostic report in *.pdf* format via the “Generate and Print Report” button, summarizing the prediction results for documentation and further analysis. While this study focuses on the fluorescence spectral range specific to breast tissue analysis, the GUI framework can be extended to accommodate other spectral data sets with appropriate model training.

## 3 Results and Discussion

This study is an attempt to elucidate the diagnosis of breast tissues as malignant and normal by native fluorescence spectroscopy. The in-house-designed and developed experimental setup was used to record native fluorescence from the tissues. The spectral processing was performed using in-house developed MATLAB codes. Minor fluctuations in the signal that were attributed to noise during signal recording were filtered using a median filter of order 10. Further, baseline correction and unity normalization were performed. A noticeable variation in the native fluorescence signal was observed in normal and malignant samples in the wavelength range from 375 to 650 nm. It was considered to be a region of interest (ROI) for further analysis.

Figure 2 shows the typical preprocessed, normalized mean native fluorescence spectra of normal and malignant breast tissues under study. The spectral peaks for both normal and malignant are due to endogenous tissue fluorophores, collagen, elastin, NADH, etc.^40^ In the case of normal cells, these peaks represent the distribution of these fluorophores in healthy tissue. However, in malignant tissue, changes in composition, metabolism, and tumor microenvironment can alter the concentration and distribution of these fluorophores, leading to differences in native fluorescence intensity and spectral characteristics compared to normal tissue. Understanding these variations may aid in diagnostic and prognostic applications in biomedical spectroscopy and imaging. The observed variations in these components hold potential as discriminative parameters for distinguishing between normal and malignant conditions. This elucidation underscores the importance of utilizing spectral characteristics as diagnostic markers in biomedical applications.^11^

**Figure 2:**
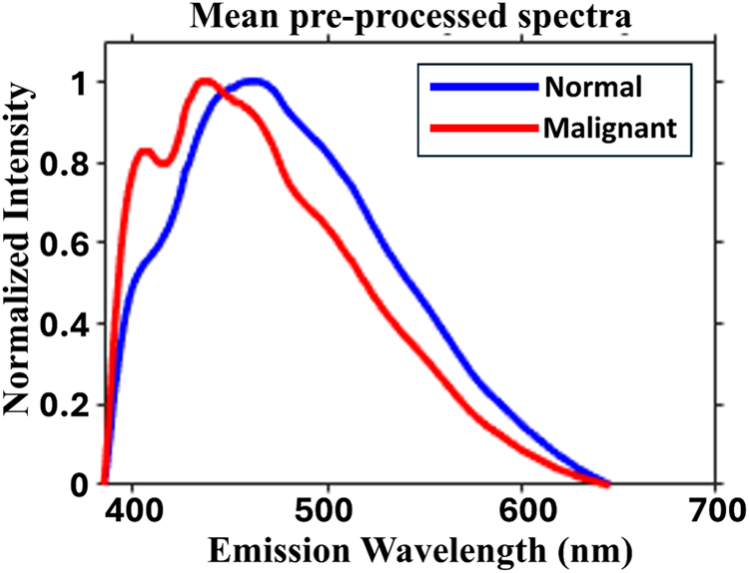
Typical preprocessed, normalized mean native fluorescence spectra of normal and malignant breast tissues.

The recorded spectra in the current study demonstrated a distinct minor peak due to collagen/elastin at ∼409 nm and a major peak due to NADH at ∼435 nm for normal tissues and at ∼409 and ∼460 nm due to collagen/elastin and NADH, respectively, for malignant tissues. In contrast, a study by *Chowdary et al.* on breast pathological tissues reported the major peak due to collagen at 390 nm, the minor peak due to NADH at ∼460 nm for malignant breast tissues, and the minor peak at ∼390 nm due to collagen and the major peak at ∼460 nm due to NADH for the normal breast tissues. However, the experimental conditions used in their study were different, used 325 nm pulsed laser light for sample excitation and a spectrograph ICCD combination for spectral recording, maybe the reason behind the variation.^11^

The recorded native fluorescence spectra were subjected to machine learning-based classification analysis. In order to improve the power of prediction ability of the machine learning model, a subset of features from the entire spectra was selected.^41^ Further, even the available features might contain redundant information, which could negatively impact the performance of the model. Therefore, in the current study, a filter-based feature selection method, the mRMR algorithm, was used to rank the most relevant features, thereby reducing the feature redundancy. There were 344 features ranked by the mRMR algorithm in the ROI under study at an increment of ∼0.78 nm. The ranking of all 344 features is shown in Figure 3, with the top 3 features highlighted in it. Feature 1 has the highest prediction score of 0.65, indicating that it is the best feature to distinguish between normal and malignant. The prediction score values for feature 2 and feature 3 were found to be 0.177 and 0.175, respectively, and thereafter, these values drastically decreased for the remaining features. These top 3 features (features 1, 2, and 3) are thus considered for further analysis in the study. Further, feature 1 corresponds to an emission wavelength of 400.6 nm, representing collagen emission, and features 2 and 3 correspond to emission wavelengths of 453.2 and 477.6 nm, respectively, representing NADH emission.^11^ Increased metabolic activity in cancer cells often leads to elevated NADH levels,^42^ while changes in the extracellular matrix can affect collagen fluorescence.^43^ These tissue fluorophores, collagen, and NADH are already known biomarkers for discriminating normal from malignant tumor tissues^11^ and are also identified by the feature selection method in the present study. Thus, this observation suggests that the mRMR is an efficient feature selection method for removing data redundancy and hence was used in the current study. The mRMR minimizes the correlation between different features of a target class and maximizes the correlation between the features and target classes. The correlation plot of the top 3 features for normal and malignant is shown in Figure 4.

**Figure 3:**
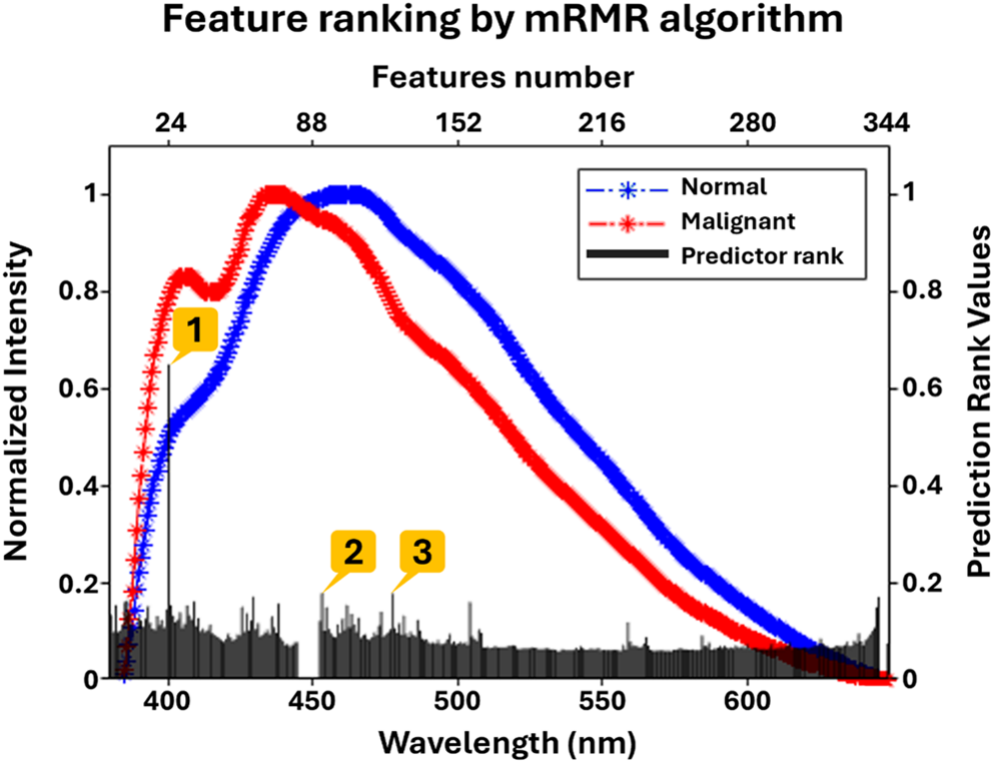
Plot showing mean native fluorescence spectra of normal and malignant breast tissues (*X*-axis-bottom wavelength vs *Y*-axis-left normalized intensity) along with the corresponding mRMR feature ranking (*X*-axis-top features number vs *Y*-axis-right prediction rank values). Highlighted numbers 1–3 correspond to the top 3 features having position (1)/feature 1: *X*-axis = 400.6 and *Y*-axis = 0.65, position (2)/feature 2: *X*-axis = 453.2 and *Y*-axis = 0.177, position (3)/feature 3: *X*-axis = 477.6 and *Y*-axis = 0.175, where the *X*-axis (bottom) corresponds to wavelength in nanometers, and the *Y*-axis on the right corresponds to prediction importance score (rank value).

**Figure 4:**
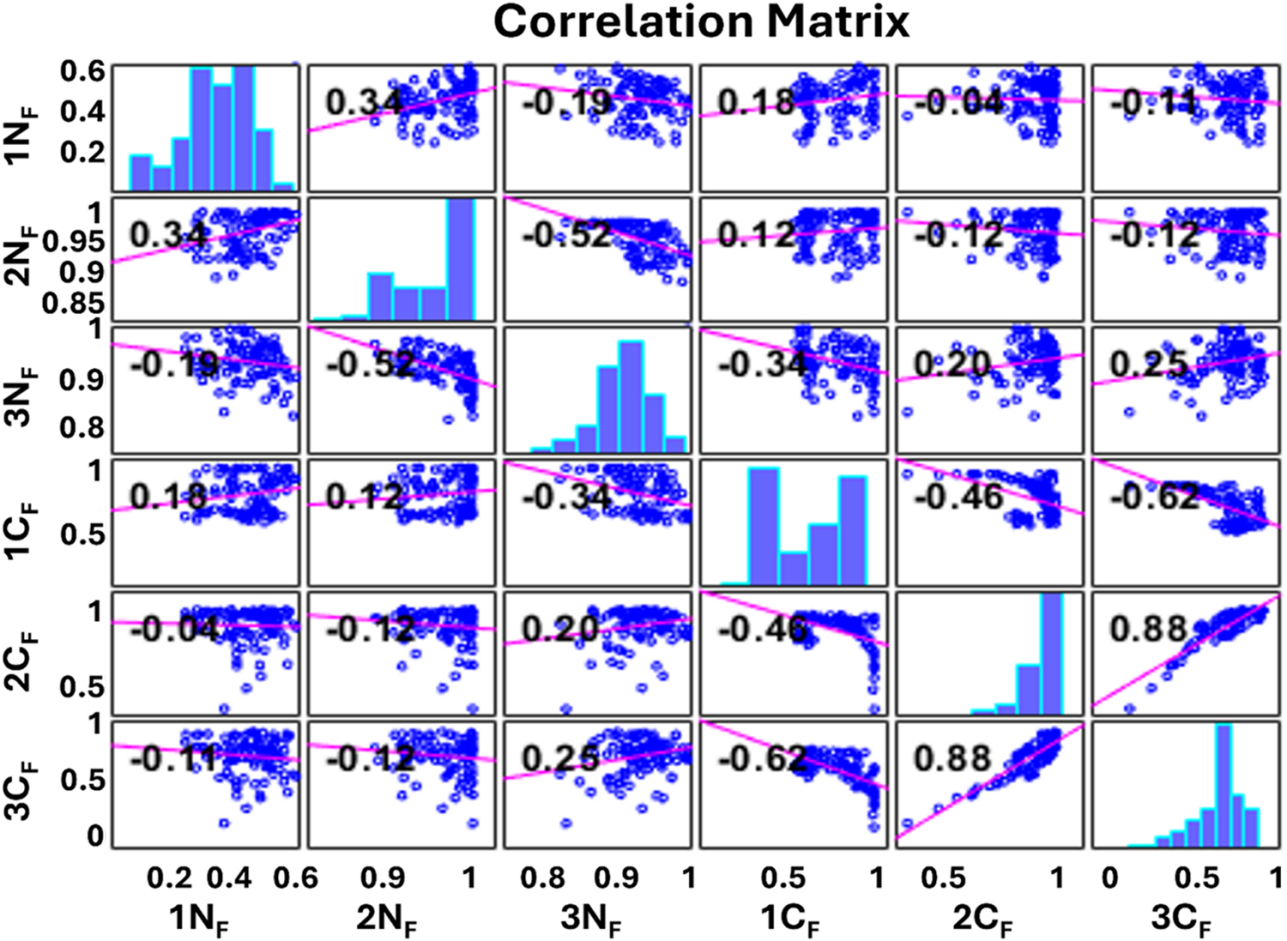
Correlation plot of the top 3 features for normal and malignant spectra. 1C_F_, 2C_F_, and 3C_F_ are the top 3 malignant features and 1N_F_, 2N_F_, and 3N_F_ are the top 3 normal features.

The correlation plot reveals that all features exhibit predominantly low correlation with each other, as indicated by the mRMR algorithm. Notably, features 2 and 3 of malignant samples demonstrate a positive correlation with an increasing feature index. Conversely, other features remain uncorrelated. Further, when scatter plots between the normalized spectral intensities of features 1, 2, and 3 versus sample numbers for each of the normalized spectra under study were plotted, they demonstrated clear discrimination between normal and malignant samples under study for feature 1 and partial discrimination for features 2 and 3, as shown in Figure 5.

**Figure 5:**
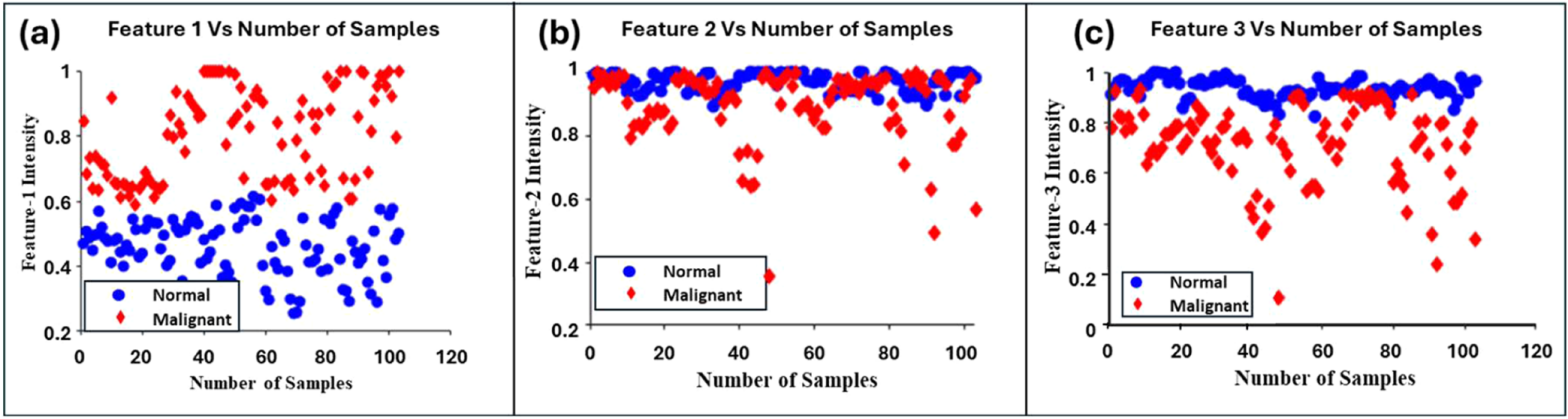
Scatter plot of top 3 features (a–c) for normal and malignant spectra against sample numbers. (a) Feature 1 versus sample number, (b) feature 2 versus sample number, and (c) feature 3 versus sample number.

Further, when the intensities of normal and malignant native fluorescence spectra for features 1, 2, and 3 in the normalized spectra were compared, they demonstrated clear variation between normal and malignant spectra, as shown in Figure 6. Mann–Whitney’s U test^44^ also showed a P-value significance of <0.0001 for all 3 features as shown in Figure 6. Further, using these intensities, intensity ratios R1 and R2 (Table 1) for each of the normal and malignant spectra under study were calculated and plotted, as shown in Figure 7. The figure clearly differentiated normal from malignant, suggesting that these 2 features were sufficient for the machine learning-based classification of the spectra under study, reducing the input feature dimension from 3 to 2. The reduced features are then used as input features for machine learning (ML) algorithms in the study. Out of 206 (103 normal, 103 malignant) spectra, 124 (62 normal, 62 malignant) spectra (60%) were used for training, and the remaining 82 (41 normal, 41 malignant) spectra (40%) were used for testing the model.

**Figure 6:**
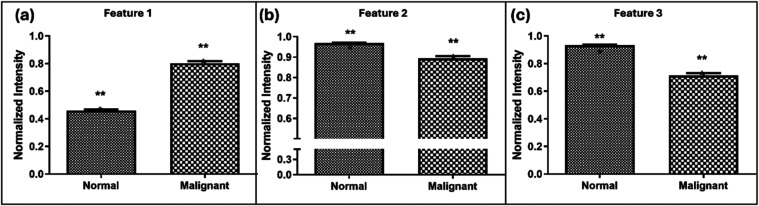
Plot of wavelength position for (a) feature 1, (b) feature 2, and (c) feature 3 in the normalized averaged native fluorescence spectra of normal and malignant samples under study (*** significance using Mann–Whitney’s U test having P < 0.0001).

**Figure 7:**
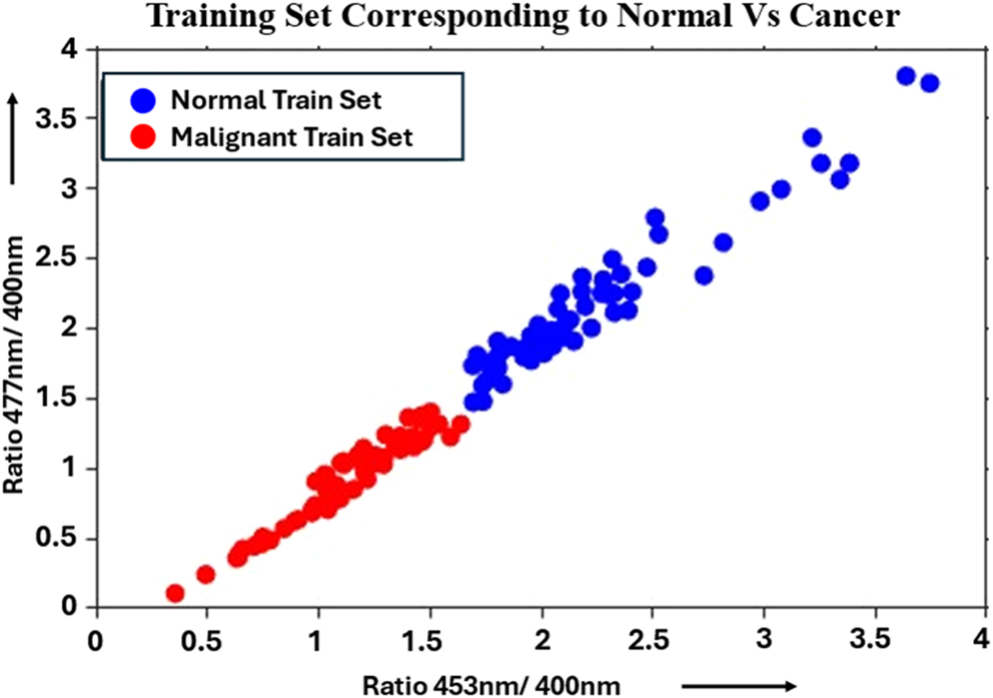
Scatter plot showing the training set corresponding to R1 versus R2 for both normal and malignant groups.

In the current study, backpropagation-ANN^45,46^ was modeled to have 3 layers, the first layer was the input layer (124 spectra × 2 intensity ratios), 1 hidden middle layer having 8 neurons, and the last layer is an output layer having 2 binary outputs corresponding to normal—0 and malignant—1. Once this model was trained, it was used to predict test data, generating prediction scores with a score value between 0 and 1. The value <0.5 was considered the normal class, and >0.5 was considered the malignant class. Upon performance evaluation, ANN-RP demonstrated an accuracy of 97.56% and sensitivity, specificity, and precision of 100, 95.12, and 95.35%, respectively. ANN-SCG^47^ demonstrated an accuracy of 98.37% and sensitivity, specificity, and precision of 100, 97.56, and 97.62%, respectively. ANN-GDA demonstrated an accuracy of 98.78% and sensitivity, specificity, and precision of 100, 97.56, and 97.62%, respectively.

Further, a support vector machine (SVM) with various kernels RBF, linear, and polynomial of order 3, was used in this study to train and test the model (124 spectra × 2 intensity ratios). The trained model was later used to calculate the prediction score values (0 for normal and 1 for malignant). In the case of SVM in different kernels, SVM-RBF demonstrated an accuracy of 97.56% and sensitivity, specificity, and precision of 100, 95.12, and 95.35%, respectively. SVM polynomial demonstrated an accuracy of 98.78% and sensitivity, specificity, and precision of 100, 97.56, and 97.62%, respectively. SVM-Linear demonstrated an accuracy of 97.56% and sensitivity, specificity, and precision of 100, 95.12, and 95.35%, respectively.

Similarly, the Naïve Bayes^48^algorithm was also designed with the same input features under study, and prediction score values of less than 0.5 were considered normal, and greater than 0.5 were considered malignant. The trained model showed an accuracy of 97.56% and sensitivity, specificity, and precision of 100, 95.12, and 95.35%, respectively. The overall accuracy, sensitivity, specificity, precision, F-score, AUC, and MCC values of the models under study are listed in Table 2.

**Table 2:**
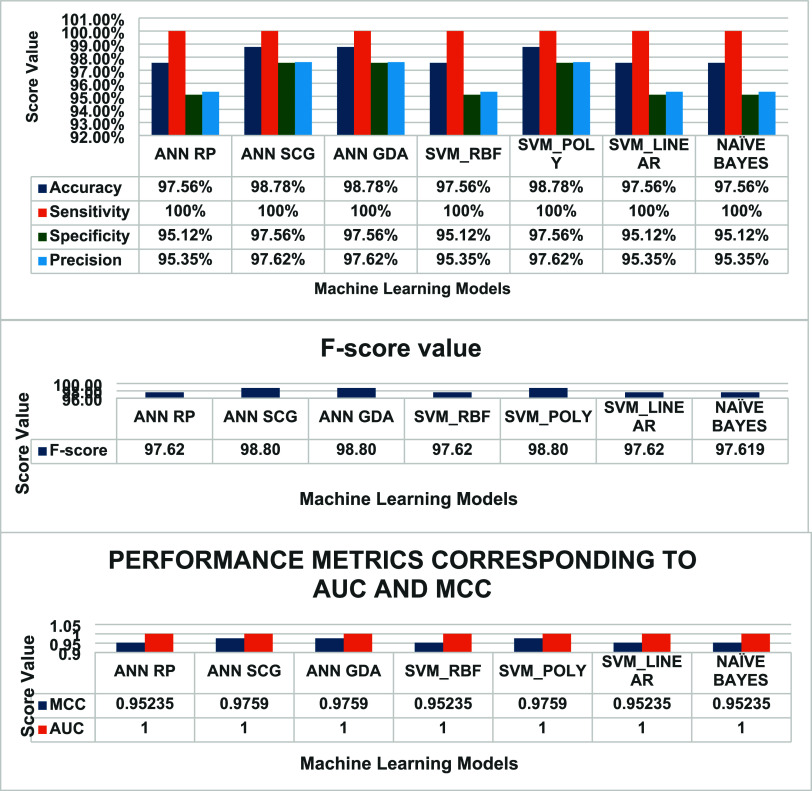
Performance Metrics Showing Accuracy, Sensitivity, Specificity, Precision, F-Score, MCC, and AUC Values for All of the ML Algorithms under Study

From Table 2, it is clear that the SVM polynomial of order 3, ANN-GDA, and ANN-SCG perform better when compared with other algorithms. This is due to the ability of polynomial kernels to model nonlinear relationships between features that may have been advantageous in capturing the complex spectral signatures associated with breast cancer, compared to linear or RBF kernels. The score values generated by the model were listed automatically in the *Excel file* in a specified folder using an in-house developed code using the MATLAB platform. Supporting Table S1 shows the score values for the samples under study and the “Match” condition in the form of “Yes/No” for the SVM polynomial kernel model. Figure 8 shows the training, testing, and classified data plotted along with the decision boundary for the SVM polynomial model. The zoomed inset shows 1 mismatched case/sample, which is actually normal but predicted as malignant.

**Figure 8:**
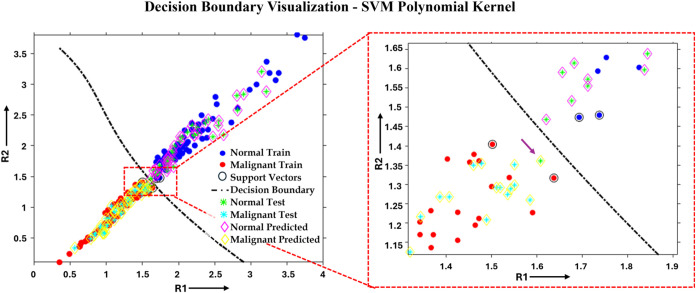
Scatter plot of training, testing, and prediction data corresponding to 2 ratios R1 and R2 for both normal and malignant classes, along with the decision boundary. The purple arrow shows the misclassification.

In the current study, 5-fold cross-validation analysis using SVM polynomial showed consistent accuracy values of 97.56, 98.78, 96.57, 98.37, and 97.09%, respectively, with an average performance of 97.67% ± 0.91%, suggesting no possibility of data overfitting and representing a generalized model. Therefore, this model was finalized as a prediction model in the back end of a graphical user interface (GUI) to classify/predict any unknown new native fluorescence spectrum of normal or malignant breast tissue under investigation on a real-time basis. Figure 9(a) represents the screenshot of the GUI consisting of a left panel with a display screen, a status bar, and a “Browse data” button. On the right side of the GUI, 2 indicators representing “Normal” and “Malignant” at the top, followed by the “Predict” and “Generate and Print Report” buttons, and an “Exit” button. When a sample data in “.spc” format is browsed, its corresponding two-dimensional (2D) spectrum gets displayed on the screen along with normalized “normal” and “malignant” spectrum presaved in the GUI as a ready reference, as shown in Figure 9(b). The file name of the selected spectrum displayed on the screen also appears on the “status bar”. After spectral display, when the “Predict” button is pressed, a green indicator light turns ON for normal (Figure 9(c)), and a red indicator light for malignant (Figure 9(d)) based on the prediction by the trained model in the back end of the GUI. A report of the prediction analysis (green/red) can be generated by pressing the “Generate and Print Report” button. This operation will provide the prediction of the loaded “.spc” file in the form of “.pdf” as a diagnosis report, as shown in Figure 9(e,f).

**Figure 9:**
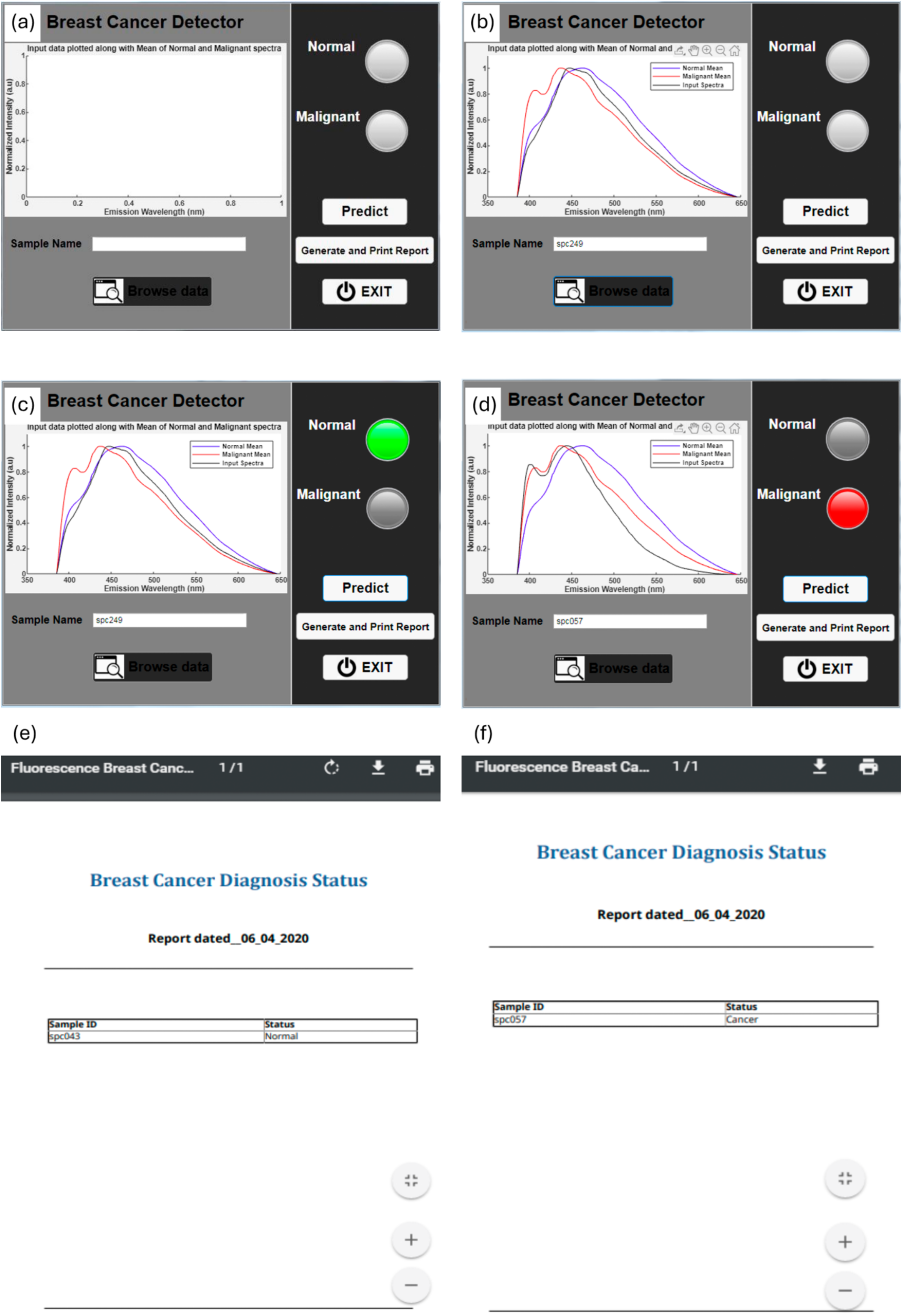
GUI for the prediction of new spectra given by the user. (a) Screenshot of the app developed. (b) Plot of the mean of normal and malignant along with the input sample. (c, d) The prediction result example for normal and malignant conditions. (e, f) The report generated with the option to print or download.

Table 3 highlights the performance of various breast cancer diagnostic techniques and compares them with the current study. Although the biopsy remains the gold standard due to its high specificity, it is invasive and time-consuming. MRI, though highly accurate, is expensive and less accessible. Ultrasound, on the other hand, is more affordable and widely available but has relatively lower sensitivity and specificity for denser breasts, making it less reliable as a standalone diagnostic tool. In contrast, our technique achieves superior diagnostic performance and offers faster results and cost-effectiveness compared to MRI and biopsy. Being noninvasive, it eliminates patient discomfort while maintaining high reliability. With its ability to provide accurate and efficient detection, our technique has strong potential for clinical translation, making it a promising alternative for early diagnosis and routine screening in healthcare settings.

**Table 3:** Comparison of Various Breast Cancer Diagnostic Modalities with the Current Study

methods	accuracy (%)	sensitivity (%)	specificity (%)	invasiveness	refs
mammography	77.9–89.3	60–97	64.5–80	noninvasive	^49–51^
ultrasound	74–85	61–87	75–76.8	noninvasive	^49,52,52^
MRI	86.9–98.4	72.2–94.6	66.7–74.2	noninvasive	^49,52^
biopsy	92.5	87–94.2	88.1–98	invasive	^53,54^
current study	98.78	100	97.56	noninvasive	

A limitation of this study is the relatively small sample size (31 normal and 31 malignant cases), which may affect the statistical robustness and generalizability of the findings, which can be overcome by including a larger sample size in future studies. Additionally, breast cancer is highly heterogeneous, with variations in molecular subtypes, metabolic profiles, and extracellular matrix composition potentially influencing the observed spectral signatures. These factors could contribute to variability in fluorescence responses, necessitating a cautious interpretation of the results.

Beyond sample size limitations, the clinical translation of fluorescence-based spectral diagnostics presents additional challenges. Larger-scale clinical trials are essential to establish the diagnostic accuracy, sensitivity, and specificity of this approach across diverse patient populations. Furthermore, regulatory approvals must be obtained to ensure compliance with clinical safety and efficacy standards. The integration of spectral-based techniques into existing diagnostic workflows also requires careful consideration, including compatibility with current imaging modalities, cost-effectiveness, and ease of use in clinical settings. Addressing these challenges will be crucial for advancing this technology toward routine clinical application and improving breast cancer detection and characterization.

## 4 Conclusions

The present study demonstrates the effectiveness of native fluorescence spectroscopy integrated with machine learning for the classification of normal and malignant breast tissues. By recording native fluorescence spectra and applying machine learning-based classification, we identified SVM polynomial as the most consistent and reliable model, achieving an accuracy of 98.78%, a sensitivity of 100%, and a specificity of 97.56% after cross-validation, ensuring a generalized model without overfitting. To translate this research into practical use, we developed a graphical user interface (GUI) based on this optimized model, enabling the real-time prediction of unknown samples in a user-friendly and time-efficient manner. This approach significantly reduces the time and effort required for fluorescence data acquisition and classification, offering a rapid, minimally invasive, and cost-effective alternative to breast cancer diagnosis. By providing accurate and immediate classification, this method has the potential to alleviate patient anxiety, expedite clinical decision-making, and reduce the economic burden associated with conventional diagnostic procedures. The integration of spectroscopy and machine learning in this study represents a promising step toward improving early breast cancer detection, facilitating timely interventions, and ultimately enhancing patient outcomes.

## Supplementary Material



## Notes

MATLAB code for implementing backpropagation artificial neural networks; support vector machines; and Naïve Bayes classifiers; extended descriptions of performance metrics; including cross-validation; confusion matrices; ROC curves; and Matthews correlation coefficient; general architecture of backpropagation artificial neural network algorithm, and score values for test data using the SVM POLYNOMIAL ML algorithm (PDF)
