# Supplementary material for: Machine Learning Empowered a Graphical User Interface on Native Fluorescence to Predict Breast Cancer
Source: ACS Omega. 2025 May 14;10(20):20315–25. doi: 10.1021/acsomega.4c11669 (PMC12120607; doi:10.1021/acsomega.4c11669)
Supplement: Supplementary file 1 [file ao4c11669_si_001.pdf]

# Machine learning empowered graphical user interface on native fluorescence to predict breast cancer

Ashwini Amin[1,2], Mallika Priya[3], Jackson Rodrigues[1], Shimul Biswas[1], Subhash Chandra[1], Stanley Mathew[4], Satadru Ray[5], Bola Sadashiva Satish Rao[6], & Krishna Kishore Mahato[1]*

*[1.] Department of Biophysics, Manipal School of Life Sciences, Manipal Academy of Higher Education, Karnataka, Manipal, India – 576104.*

*[2.] Department of Computer Science & Engineering, Manipal Institute of Technology, Manipal Academy of Higher Education, Karnataka, Manipal, India – 576104.*

*[3.] Clinical Mind, Boston, Massachusetts, United States-02108*

*[4.] Department of General Surgery, Kasturba Medical College, Manipal Academy of Higher Education, Karnataka, Manipal, India – 576104.*

*[5.] Department of General Surgery, Kasturba Medical College, Manipal Academy of Higher Education, Karnataka, Mangaluru, India – 575001.*

*[6.] Department of Radiation Biology & Toxicology, Manipal School of Life Sciences, Manipal Academy of Higher Education, Karnataka, Manipal, India – 576104.*

** Corresponding Author, Email – mahato.kk@manipal.edu; kkmahato@gmail.com*

## 1. METHODS

### Data Analysis

### *Machine Learning*

Machine learning is a hot research area in the field of sciences, which is described as “precision medicine” in the field of biomedical engineering. It's a framework that takes in the information, identifies patterns, trains itself to utilize the information, and yields a result. This has many advantages when compared to humans doing the same work. Machines can work faster than humans and never get exhausted. After every iteration machine will perform better and the precision of a machine is more when compared to the human. There are a variety of machine learning algorithms that are trending these days.

### *Backpropagation Artificial Neural Networks.*

This type of network is in trend when compared to feed-forward networks because the error-correcting capability of these networks is very high when compared to feed-forward networks. The backpropagation learning consists of 2 passes which are forward and backward. In the case of the forward pass, applied input will move layer by layer, outputs are generated as genuine reactions, moreover, weights are set. Weights are modified in the event of a backward pass by applying the rule of error correction. The error signals are obtained by taking the difference between the actual response and desired response. This is then backpropagated using the network. There are many algorithms available in ANN like gradient descent, gradient descent with adaptive learning rate, Resilient backpropagation algorithm, and scaled conjugate gradient [2], [3], [4]

For our study, Backpropagation ANN is made of three layers: 1st is input layer,2nd is hidden layer with 8 neurons, and 3rd is the output layer. The architecture is as shown in Fig. S1 Input layer has 2 neurons as it takes 2 inputs and the output layer consisting of 1 neuron produces 1 output in terms of binary 0 or 1. The hidden layer was designed with 8 neurons based on trial and error for the best outcome.

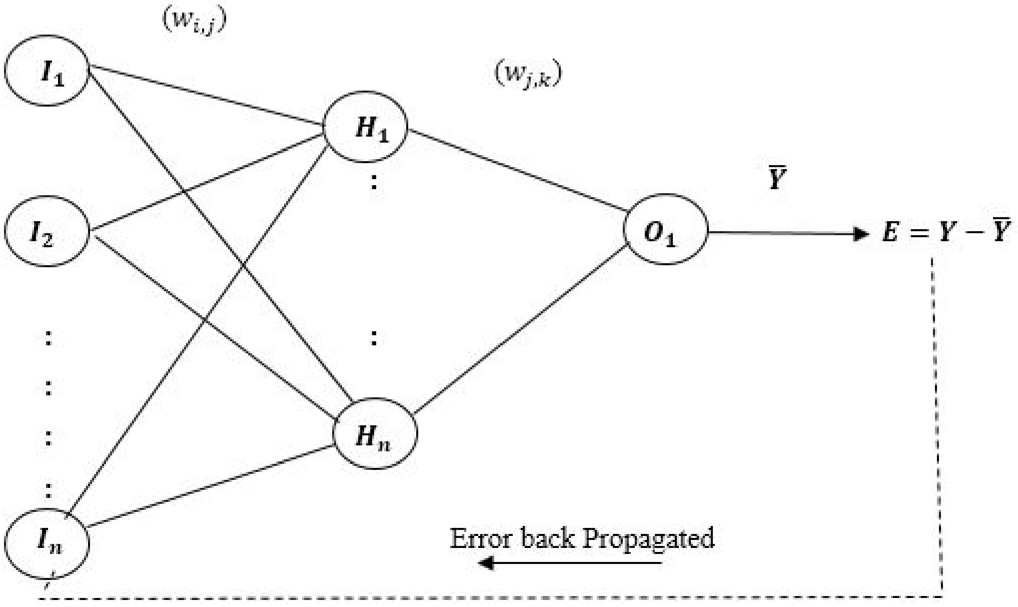


Input Layer (n =2) Hidden Layer(s) (n =8) Output Layer (n =1)

**Fig. S1** General architecture of backpropagation artificial neural network algorithm.

Here $(I_1, I_2, .... I_n)$ are input layer neurons, $(H, H_2, .... H_n)$ are hidden layer neurons and $O_1$ is the output layer neuron. E determines the error difference in the estimated output $\overline{Y}$ and desired output Y which is back propagated as shown in Fig. S1. The weights $(w_{i,j})$ represent weight in comparison to hidden layer and input layer and $(w_{j,k})$ weight corresponding to output layer and hidden layer.

Every neuron from each layer is linked to the neurons in another layer using weights $(w_{i,j})$ and $(w_{j,k})$. The neurons calculate the weighted sum based on the activation function in each neuron for every layer. In our study 'tansig' is used as the activation function since the expression below indicates that it produced superior outcomes when compared to other activation functions.

$$f(x) = \frac{2}{(1 + e^{-2n}) - 1}, where\ n\ represtents\ the\ net\ input\ vector$$

After the activation process continues up to the output layer the estimated output is calculated and further compared with the desired target value. The resulting value of the error which is the desired value subtracted from the predicted value is propagated back to the network so that weights get

modified and eventually the error also reduces. This process is known as the training of the network. Once the model is trained this can be used to predict the unknown test data.

### *2.3.2. Support vector machine*

These algorithm works with both linear as well as nonlinear classes of data. Usually, SVM is used for binary classification, but this can be extended for multi-class classification as well. The algorithm separates classes through a hyperplane, which is determined by margins and support vectors. The margin represents the maximum distance between the hyperplane and the nearest data points of different classes. The data points closest to the hyperplane are known as support vectors. and any change in these points alters the hyperplane's position. Various kernel functions can be used, such as linear, Radial Basis Function (RBF), sigmoid and polynomial to map the data into different dimensions for better separation. Based on the choice of the kernels the SVM models are trained. Two hyperparameters need to be tuned while training for getting better accuracy values which is done by making the generalization error as low as possible. These parameters are known as regularization parameters C and gamma (γ) and these hyperparameters are usually tuned in logscales. The smaller the value γ more the effect of the single sample and the boundary will become straight and the larger the value γ more detailed the separation, but this will overfit the data and hence generalization will be bad. C is the amount of penalty added when we misclassify the data. It ignores the points near to boundary and hence increases the value of the margin and hence the separation. Large C means linear will be the boundary line and small C means the boundary line will be detailed. The bias values should be low to ensure there is good training accuracy. For the polynomial kernel, the accuracy increases as the degree of the polynomial increases because the boundary line becomes more curved [5], [6].

Let us consider the feature vector for classification given by the below equation

$$X = \{\bar{x}_1, \bar{x}_2 \ldots\ldots, \bar{x}_n\} where\ \bar{x}_i \in \mathbb{R}^m$$

Binary class targets are denoted by the below equation

$$Y = \{y_1, y_2 \ldots\ldots, y_n\}\ where\ y_n \in \{-1, 1\}$$

The hyperplane equation is given below for the linear case

$$\bar{w}^T\bar{x} + b = 0\ \ where\ \bar{w} = \begin{pmatrix} w_1 \\ \cdot \\ \cdot \\ w_m \end{pmatrix}\ and\ \bar{x} = \begin{pmatrix} x_1 \\ \cdot \\ \cdot \\ x_m \end{pmatrix}$$

Therefore, the output of the classifier is given by

$$\hat{y} = f(\bar{x}) = sgn(\bar{w}^T\bar{x} + b)$$

For the binary classification, the hyperplane equations are given below

$$\begin{Bmatrix} \overline{w}^T\overline{x} + b = -1 \\ \overline{w}^T\overline{x} + b = 1 \end{Bmatrix}$$

For generating flexible hyperplanes in the case of a nonlinear classifier below will be the constraint.

$$y_i(\overline{w}^T\overline{x} + b) \geq 1 - \zeta_i$$

Where $\zeta_i$ is known as the slack variable which is used to generate flexible margins. For the nonlinear-based classification, we need to project the data to a higher dimension where the data can be linearly separated, and this technique is called as the kernel trick. [7] The general equation is shown below

$$K(\overline{x}_i, \overline{x}_j) = \phi(\overline{x}_i)^T\phi(\overline{x}_j)$$

For RBF and polynomial type, the kernel equation is as shown below

$$\text{RBF } : K(\overline{x}_i, \overline{x}_j) = e^{-\gamma|\overline{x}_i - \overline{x}_j|^2}$$

Polynomial: $K(\overline{x}_i, \overline{x}_j) = (\gamma \overline{x_i}^T x_j + r)^d$ where d is the degree of the polynomial

Using the trained model prediction can be done to obtain the classification score.

In SVM, the score used to classify a sample value (x) is the signed distance from x to the decision boundary, ranging from -∞ to +∞. A positive score indicates the sample belongs to one class, while a negative score suggests it belongs to another. The predicted score is calculated using a function involving SVM parameters $(\alpha_1, \alpha_2, \ldots, \alpha_n, b)$ obtained from the trained model, with G ($x_j$, $x$) representing the predictor region's dot product of the x and support vectors [8].

$$\hat{f}(x) = \sum_{j=1}^{n} \alpha_j y_j G(x, x_j) + \hat{b}$$

### *2.3.3. Naïve Bayes.*

Naive Bayes is a powerful group of classifiers that decide the likelihood of a result given under a set of conditions by using Bayes' theorem [9]. It is based on the probabilistic approach. One of the disadvantages of this classifier is that it assumes the input data to be normally distributed but in many real-time scenarios, this is not the case [10].

Let us consider the dataset for classification given by the below equation

$$X = \{\overline{x}_1, \overline{x}_2 \ldots\ldots, \overline{x}_n\} where\ \overline{x}_i \in \mathbb{R}^m$$

And every feature vector be represented as: $\overline{x}_\mathrm{i} = [x_1, x_2, \ldots\ldots, x_m]$

Let the target dataset be represented as shown below

$Y = \{y_1, y_2 ......, y_n\}$ $where\ y_n \in \{0,1,2,3,...P\}$ where P is the number of classes

Considering Bayes' Theorem, the predicted class is obtained by the posterior probability which has the maximum value for the class it belongs to is given by the below equation.

$$P(y \mid x_1, x_2, ..., x_m) = \alpha P(y) \prod_i P(x_i | y)$$

Where P(y) is the Apriori probability and $P(x_i|y)$ is the conditional probability of $x_i$ given y

## *2.4 Performance Metrics.*

There are many ways in which the performance of the algorithm can be evaluated to achieve specific objectives like explained below

### *2.4.1 Cross- Validation.*

This is the most commonly used method to estimate how the model performs in general to the entire dataset. Particularly, K-fold cross validation approach is used. Here the idea of this approach is used to divide entire dataset X into a training and test set (the remaining part). The size of the test data is determined by the number of folds.

Below are the steps performed using this approach:

1. Randomly shuffle the dataset.
2. The dataset is divided into k groups. Here in the above example k =5.
3. For each iteration test data set is taken out and the remaining groups will be the training data set. In the example above 1 set is test set and remaining are the train sets.
4. The model is fit and trained, and evaluation is performed on the test set
5. The evaluation score is stored, and the model is discarded

After all the iterations, the skill of the model is summarized using the evaluation scores. By using this approach, it's possible to find out the accuracy of the model using different sampling ratios. Ideally, the accuracy obtained in each iteration should be very close to each other [11].

### *2.4.2. Confusion Matrix.*

This is a table (as shown in Fig. S2) that is often used to report the performance of a classification model. This report is based on a set of test data for which the target values are known [12].

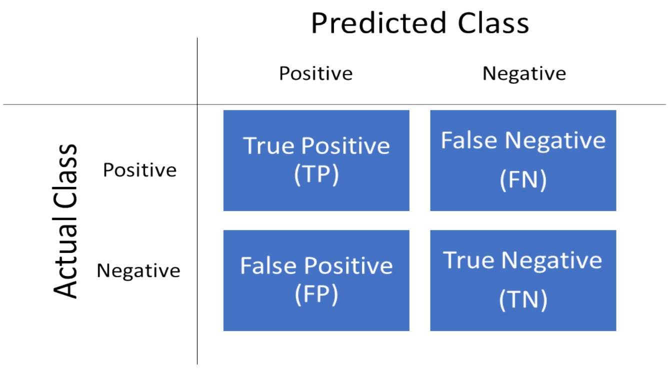


**Fig. S2** General form of Confusion Matrix of Predicted Class Versus Actual Class.

True +Ve (TP): A +Ve instance correctly identified as +Ve.

False +Ve (FP): A -Ve instance incorrectly identified as +Ve.

True -Ve (TN): A -Ve instance correctly identified as -Ve.

False Negative (FN): A +Ve instance incorrectly identified as -Ve.

From the confusion matrix below parameters can be calculated:

- Accuracy: It is a measure of how often the classifier correctly classifies the data.

$$\frac{TP + TN}{TP + TN + FP + FN}$$

- Sensitivity: Also referred to as the true positive rate or recall, it measures how frequently the classifier correctly predicts a positive outcome when the actual condition is positive.

$$\frac{TP}{TP + FN}$$

- Specificity: It is known as the true -Ve rate, which measures how often the classifier correctly predicts "no" when the actual condition is "no."

$$\frac{TN}{FP + TN}$$

- Precision: This measures the classifier's accuracy in predicting correct outcomes and reflects its ability to identify positive samples accurately and minimize misclassification.

$$\frac{TP}{FP + TP}$$

- $F_{beta}$ : When beta is set to 1, it calculates the F1 score, which is the weighted harmonic mean of precision and recall. When the value of beta is less than 1 then more importance is given to precision and if the value is greater than 1 then more importance is given to recall.

$$F_{beta} = (\beta^2 + 1)\frac{Precision\,.\,Recall}{(\beta^2\ Precision) + Recall}$$

$$F_1 = 2\frac{Precision\,.\,Recall}{Precision + Recall}$$

***2.4.3. ROC Curve***

A graphical representation called the Receiver Operating Characteristic (ROC) curve is used to assess how well a classification model is doing. The Area Under the Curve (AUC) quantifies how well the model distinguishes between different classes, with a higher AUC indicating a more accurate predictive capability. In the ROC plot (illustrated in Fig. S3), the x-axis depicts the false positive rate (or specificity), while the y-axis shows the true positive rate (or sensitivity) [13].

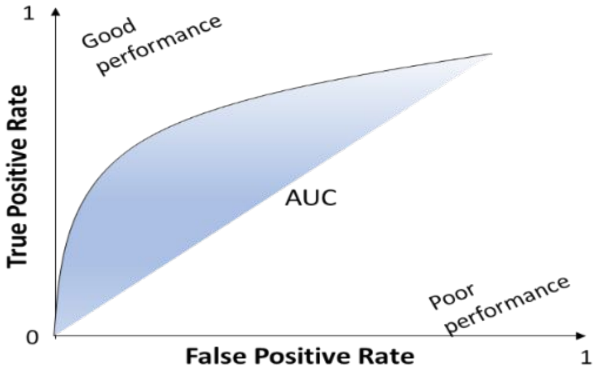


**Fig. S3** ROC (Receiver Operating Characteristic) curve of True positive rate versus False positive rate.

2.4.4. Mathew's correlation coefficient.

Even though the value of Accuracy and F1 score which is computed from the confusion matrices are amongst the most popular metrics adopted in binary classification problems however they may show overoptimistic results mostly on the imbalanced datasets. Matthews correlation coefficient (MCC) offers a more dependable metric, providing a high score only when the true +Ve, true -Ve, false -Ve, and false +Ve are all well-balanced and yield strong results [14].

**Table S1**

Score values for test data using the SVM POLYNOMIAL ML algorithm. Binary class 0 corresponds to Normal Condition and 1 corresponds to Malignant Condition. Match 'Yes' indicates that both the Predicted and Desired class belongs to same class.

| Sl. No. | Score Value | Predicted | Desired | Match(Y/N) |
|---|---|---|---|---|
| 1 | -13.9075 | 0 | 0 | Yes |
| 2 | -16.7186 | 0 | 0 | Yes |
| 3 | -5.6588 | 0 | 0 | Yes |
| 4 | -8.57466 | 0 | 0 | Yes |
| 5 | -19.9846 | 0 | 0 | Yes |
| 6 | -17.2015 | 0 | 0 | Yes |
| 7 | -5.47006 | 0 | 0 | Yes |
| 8 | -4.2605 | 0 | 0 | Yes |
| 9 | -56.7081 | 0 | 0 | Yes |
| 10 | -6.36702 | 0 | 0 | Yes |
| 11 | -6.44755 | 0 | 0 | Yes |
| 12 | -14.2297 | 0 | 0 | Yes |
| 13 | -8.72276 | 0 | 0 | Yes |
| 14 | -23.8263 | 0 | 0 | Yes |
| 15 | -20.2445 | 0 | 0 | Yes |
| 16 | -20.8118 | 0 | 0 | Yes |
| 17 | -23.8949 | 0 | 0 | Yes |
| 18 | -37.5287 | 0 | 0 | Yes |
| 19 | -1.96027 | 0 | 0 | Yes |
| 20 | -6.95601 | 0 | 0 | Yes |
| 21 | -1.31811 | 0 | 0 | Yes |
| 22 | -5.1153 | 0 | 0 | Yes |
| 23 | -2.25061 | 0 | 0 | Yes |
| 24 | -2.03747 | 0 | 0 | Yes |
| 25 | -0.35862 | 0 | 0 | Yes |
| 26 | -4.93572 | 0 | 0 | Yes |
| 27 | 0.794986 | 1 | 0 | No |
| 28 | -25.8154 | 0 | 0 | Yes |
| 29 | -18.3753 | 0 | 0 | Yes |
| 30 | -12.5263 | 0 | 0 | Yes |
| 31 | -27.9022 | 0 | 0 | Yes |
| 32 | -3.67215 | 0 | 0 | Yes |
| 33 | -26.0214 | 0 | 0 | Yes |
| 34 | -5.92568 | 0 | 0 | Yes |
| 35 | -3.83902 | 0 | 0 | Yes |
| 36 | -2.4621 | 0 | 0 | Yes |
| 37 | -17.3433 | 0 | 0 | Yes |

| | | | | |
|---|---|---|---|---|
| 38 | -44.3876 | 0 | 0 | Yes |
| 39 | -47.2991 | 0 | 0 | Yes |
| 40 | -66.6748 | 0 | 0 | Yes |
| 41 | -10.2853 | 0 | 0 | Yes |
| 42 | 6.561484 | 1 | 1 | Yes |
| 43 | 1.927562 | 1 | 1 | Yes |
| 44 | 4.495429 | 1 | 1 | Yes |
| 45 | 1.914855 | 1 | 1 | Yes |
| 46 | 5.179004 | 1 | 1 | Yes |
| 47 | 1.770099 | 1 | 1 | Yes |
| 48 | 4.623507 | 1 | 1 | Yes |
| 49 | 3.073279 | 1 | 1 | Yes |
| 50 | 2.923591 | 1 | 1 | Yes |
| 51 | 1.876079 | 1 | 1 | Yes |
| 52 | 1.289088 | 1 | 1 | Yes |
| 53 | 2.078559 | 1 | 1 | Yes |
| 54 | 2.036052 | 1 | 1 | Yes |
| 55 | 6.374314 | 1 | 1 | Yes |
| 56 | 7.231223 | 1 | 1 | Yes |
| 57 | 6.280923 | 1 | 1 | Yes |
| 58 | 7.990042 | 1 | 1 | Yes |
| 59 | 6.54552 | 1 | 1 | Yes |
| 60 | 6.134719 | 1 | 1 | Yes |
| 61 | 4.732451 | 1 | 1 | Yes |
| 62 | 8.273974 | 1 | 1 | Yes |
| 63 | 7.491446 | 1 | 1 | Yes |
| 64 | 1.758498 | 1 | 1 | Yes |
| 65 | 6.258153 | 1 | 1 | Yes |
| 66 | 5.517571 | 1 | 1 | Yes |
| 67 | 8.472805 | 1 | 1 | Yes |
| 68 | 8.451425 | 1 | 1 | Yes |
| 69 | 8.318793 | 1 | 1 | Yes |
| 70 | 8.415696 | 1 | 1 | Yes |
| 71 | 4.406428 | 1 | 1 | Yes |
| 72 | 3.711429 | 1 | 1 | Yes |
| 73 | 2.953963 | 1 | 1 | Yes |
| 74 | 2.08929 | 1 | 1 | Yes |
| 75 | 8.680443 | 1 | 1 | Yes |
| 76 | 7.807143 | 1 | 1 | Yes |
| 77 | 9.018635 | 1 | 1 | Yes |
| 78 | 8.812734 | 1 | 1 | Yes |
| 79 | 8.130159 | 1 | 1 | Yes |
| 80 | 7.279597 | 1 | 1 | Yes |
| 81 | 5.753905 | 1 | 1 | Yes |

| | | | | |
|---|---|---|---|---|
| 82 | 9.616805 | 1 | 1 | Yes |

5-fold Cross-validation shows accuracy values of 97.56%, 98.78%, 96.57%, 98.37%, and 97.09% respectively and these values are very close to each other. Therefore, our model is generalized well and there is no possibility of overfitting.

**MATLAB Code :**

### Code for feature extraction of fluorescence signal:

```
global X_Axis;global X_Axis_Full;%%% Read the input spc file for both Cancer
and NormalNormalDataPath = &#39;E:\Ashwini\data\Breast Cancer 325nm Flouroscence
- Latest\Normal&#39;;CancerDataPath = &#39;E:\Ashwini\data\Breast Cancer 325nm
Flouroscence - Latest\Cancer&#39;; % Excel file to store the data yourFolder =
&#39;E:\Ashwini\Matlab\Results\&#39;;newSubFolder =
[yourFolder,datestr(now,&#39;yyyy_mm_dd&#39;)];if ~exist(newSubFolder,
&#39;dir&#39;)
mkdir(newSubFolder);endfilename =
[newSubFolder,&#39;\&#39;,datestr(now,&#39;yyyymmdd_HHMM&#39;),&#39;Floro_Inp
ut_NC.xlsx&#39;];filenam
eReport =
[newSubFolder,&#39;\&#39;,datestr(now,&#39;yyyymmdd_HHMM&#39;),&#39;Floro_Inp
ut_NC&#39;]; %
Read Normal folder files D_main_Normal = dir( NormalDataPath ) ;
D_main_Normal = D_main_Normal(3:end) ;
preProcessed_N=cell(1,length(D_main_Normal));
rawData_N=cell(1,length(D_main_Normal)); for dId = 1 : numel( D_main_Normal
) D_sub1 = dir( fullfile( NormalDataPath, D_main_Normal(dId).name, &#39;*&#39; ))
```

```
; D_sub1 = D_sub1(3:end) ; n1Files = numel( D_sub1 ) ; subMean_1 =
cell(1,n1Files); for sID = 1: n1Files D_sub = dir( fullfile(
NormalDataPath, D_main_Normal(dId).name,D_sub1(sID).name, &#39;**&#39; )) ; D_sub
= D_sub(3:end) ; nFiles = numel( D_sub ) ; NormalRaw=cell(1,nFiles);
for fId = 1 : nFiles file_nm =fullfile( NormalDataPath,
D_main_Normal(dId).name,D_sub1(sID).name, D_sub(fId).name); NormalrawFile
= tgspcread(file_nm); NormalRaw{fId}(:,:,1)= NormalrawFile.Y; end
mean_P_raw = mean(cell2mat(NormalRaw)&#39;)&#39;; data_Trunc =
mean_P_raw(227:570,:); X_Axis = NormalrawFile.X(227:570,:); X_Axis_Full =
NormalrawFile.X; bkgsize=round(length(X_Axis)/10); lxx=length(X_Axis);
XX1=X_Axis(1:round(lxx/bkgsize)); XX2=X_Axis((lxx-
round(lxx/bkgsize)+1):lxx);
Y1=data_Trunc(1:(round(length(X_Axis)/bkgsize))); Y2=data_Trunc((lxx-
round(lxx/bkgsize)+1):lxx); bkgcoef=polyfit([XX1,XX2],[Y1,Y2],1);
bkg=polyval(bkgcoef,X_Axis); baselineCorrected=data_Trunc-bkg; filtered
= medfilt1(baselineCorrected,10); Area=trapz(X_Axis,filtered);
filtered=filtered./Area; meanPositionData = filtered ./ max(filtered(:));
subMean_1{sID}(:,:,1) = meanPositionData; raw_Sub{sID}(:,:,1) =
mean_P_raw; end preProcessed_N{dId}(:,:,1) = subMean_1;
rawData_N{dId}(:,:,1) = raw_Sub;end%Read the files from Cancer folder
D_main_Cancer = dir( CancerDataPath ) ; D_main_Cancer = D_main_Cancer(3:end);
preProcessed_C=cell(1,length(D_main_Cancer));
rawData_C=cell(1,length(D_main_Cancer));for dId = 1 : numel( D_main_Cancer )
D_sub1 = dir( fullfile( CancerDataPath, D_main_Cancer(dId).name, &#39;*&#39; )) ;
D_sub1 = D_sub1(3:end) ; n1Files = numel( D_sub1 ) ; subMean_1 =
```

cell(1,n1Files); for sID = 1: n1Files D_sub = dir( fullfile( CancerDataPath, D_main_Cancer(dId).name,D_sub1(sID).name, '**' )) ; D_sub = D_sub(3:end) ; nFiles = numel( D_sub ) ; CancerRaw=cell(1,nFiles); for fId = 1 : nFiles file_nm =fullfile( CancerDataPath, D_main_Cancer(dId).name,D_sub1(sID).name, D_sub(fId).name); CancerrawFile = tgspcread(file_nm); CancerRaw{fId}(:,:,1)= CancerrawFile.Y; end mean_P_raw = mean(cell2mat(CancerRaw)')'; data_TruncC = mean_P_raw(227:570,:); bkgsize=round(length(X_Axis)/10); lxx=length(X_Axis); XX1=X_Axis(1:round(lxx/bkgsize)); XX2=X_Axis((lxx-round(lxx/bkgsize)+1):lxx); Y1=data_TruncC(1:(round(length(X_Axis)/bkgsize))); Y2=data_TruncC((lxx-round(lxx/bkgsize)+1):lxx); bkgcoef=polyfit([XX1,XX2],[Y1,Y2],1); bkg=polyval(bkgcoef,X_Axis); baselineCorrected=data_TruncC-bkg; filtered = medfilt1(baselineCorrected,10); Area=trapz(X_Axis,filtered); filtered=filtered./Area; meanPositionData = filtered ./ max(filtered(:)); subMean_1{sID}(:,:,1) = meanPositionData; raw_Sub{sID}(:,:,1) = mean_P_raw; end preProcessed_C{dId}(:,:,1) = subMean_1; rawData_C{dId}(:,:,1) = raw_Sub;end Processed_Normal = preProcessed_N;while any(cellfun(@iscell,Processed_Normal)) Processed_Normal = [Processed_Normal{cellfun(@iscell,Processed_Normal)} Processed_Normal(~cellfun(@iscell,Processed_Normal))];end Processed_Cancer = preProcessed_C;while any(cellfun(@iscell,Processed_Cancer)) Processed_Cancer = [Processed_Cancer{cellfun(@iscell,Processed_Cancer)} Processed_Cancer(~cellfun(@iscell,Processed_Cancer))];end ProccesedrawData_N = rawData_N;while any(cellfun(@iscell,ProccesedrawData_N))

```
ProccesedrawData_N = [ProccesedrawData_N{cellfun(@iscell,ProccesedrawData_N)}
ProccesedrawData_N(~cellfun(@iscell,ProccesedrawData_N))];end
ProccesedrawData_C = rawData_C;while any(cellfun(@iscell,ProccesedrawData_C))
ProccesedrawData_C = [ProccesedrawData_C{cellfun(@iscell,ProccesedrawData_C)}
ProccesedrawData_C(~cellfun(@iscell,ProccesedrawData_C))];end %%
figureplot(X_Axis,preProcessed_N{172},&#39;b&#39;,&#39;Linewidth&#39;,2); hold on;
plot(X_Axis,preProcessed_C{190},&#39;r&#39;,&#39;Linewidth&#39;,2);title(&#39;Pre
Processed-
Filtered, baseline Corrected , Normalized and ROI (350nm -
650nm)&#39;)xlabel(&#39;Wavelength in nm&#39;)ylabel(&#39;Normalized
Intensity&#39;)legend(&#39;Normal&#39;,&#39;Cancer&#39;) %% Preprocessed data plot
of all samples
figure();for i = 1:length(Processed_Cancer)
plot(X_Axis,Processed_Normal{i},&#39;b&#39;); hold on
plot(X_Axis,Processed_Cancer{i},&#39;r&#39;);endylim([0
1.1])title(&#39;\fontname{times
New Roman} \bf All spectras Pre-
processed&#39;,&#39;FontSize&#39;,10)xlabel(&#39;\fontname{times New Roman} \bf
Emission
Wavelength (nm)&#39;,&#39;FontSize&#39;,10)ylabel(&#39;\fontname{times New
Roman} \bf
Normalized Intensity
(a.u)&#39;,&#39;FontSize&#39;,10)legend(&#39;Normal&#39;,&#39;Malignant&#39;)
hold
```

```
offset(gca,&#39;TickDir&#39;,&#39;out&#39;);set(gca,&#39;FontWeight&#39;,&#39;bold
&#39;)%% Raw data plot of
all samples figure();for i = 1:length(ProccesedrawData_C)
plot(X_Axis_Full,ProccesedrawData_N{i},&#39;b&#39;); hold on
plot(X_Axis_Full,ProccesedrawData_C{i},&#39;r&#39;);end title(&#39;\fontname{times
New
Roman} \bf All Raw Spectras
Recorded&#39;,&#39;FontSize&#39;,12)xlabel(&#39;\fontname{times New Roman} \bf
Emission Wavelength
(nm)&#39;,&#39;FontSize&#39;,12)ylabel(&#39;\fontname{times New Roman} \bf
Intensity
(a.u)&#39;,&#39;FontSize&#39;,12)legend(&#39;Normal&#39;,&#39;Malignant&#39;)hol
d
offset(gca,&#39;TickDir&#39;,&#39;out&#39;);set(gca,&#39;FontWeight&#39;,&#39;bold
&#39;)%% Mean of all the
raw spectrameanN_Raw = mean(cell2mat(ProccesedrawData_N)&#39;);meanC_Raw =
mean(cell2mat(ProccesedrawData_C)&#39;); %% data_Trunc =
DayCRaw.Y(227:570,:);meanN_Raw_new = meanC_Raw(:,227:570)&#39;;
bkgsize=round(length(X_Axis)/10); lxx=length(X_Axis);
XX1=X_Axis(1:round(lxx/bkgsize)); XX2=X_Axis((lxx-
round(lxx/bkgsize)+1):lxx);
Y1=meanN_Raw_new(1:(round(length(X_Axis)/bkgsize)));
Y2=meanN_Raw_new((lxx-round(lxx/bkgsize)+1):lxx);
bkgcoef=polyfit([XX1,XX2],[Y1,Y2],1); bkg=polyval(bkgcoef,X_Axis);
baselineCorrected=meanN_Raw_new-bkg; filtered =
```

medfilt1(baselineCorrected,10); Area=trapz(X_Axis,filtered);
filtered=filtered./Area; meanC_Raw_processed = filtered ./
max(filtered(:)); %%cont = cell2mat(Processed_Normal)';contCatN =
horzcat(cont,zeros((size(cell2mat(Processed_Normal),2)),1));cont =
cell2mat(Processed_Cancer)';contCatC =
horzcat(cont,ones((size(cell2mat(Processed_Cancer),2)),1));AllData =
vertcat(contCatN, contCatC);% xlswrite(filename,header,'AllData');%
xlswrite(filename,AllData,'AllData',sprintf('A2')); fs_X =
AllData(:,1:end-
1);fs_Y = AllData(:,end);[idx,scoresMrmr] = fscmrmr(fs_X,fs_Y); % colNames1 =
cellstr( num2str( (1:size(cell2mat(preProcessed_N),1)).',
'F_%d' ) ) .';%
colNames_Mat = cell2mat(colNames1)% colNames =
convertCharsToStrings(colNames_Mat)% AllData_Table =
array2table(FeaturesSelected,'VariableNames',colNames);
bar(scoresMrmr(idx))scores_edit = scoresMrmr; xlabel('\fontname{times New
Roman} \bf Predictor Index value corresponding to highest
score','FontSize',12)ylabel('\fontname{times New Roman} \bf
Predictor
importance score','FontSize',12)xticklabels(idx)title('\fontname{times
New
Roman} \bf Feature ranking based on MRMR Feature Selection
Algorithm','FontSize',12) X_AxisLoc1 = X_Axis(idx(1));X_AxisLoc2 =
X_Axis(idx(2));X_AxisLoc3 = X_Axis(idx(3));X_AxisLoc4 =
X_Axis(idx(4));X_AxisLoc5 = X_Axis(idx(5));X_AxisLoc6 =

```
X_Axis(idx(6));X_AxisLoc7 = X_Axis(idx(7));X_AxisLoc8 =
X_Axis(idx(8));X_AxisLoc9 = X_Axis(idx(9));X_AxisLoc10 =
X_Axis(idx(10));feature1 = AllData(:,idx(1));feature2 =
AllData(:,idx(2));feature3 = AllData(:,idx(3));feature4 =
AllData(:,idx(4));feature5 = AllData(:,idx(5));feature6 =
AllData(:,idx(6));feature7 = AllData(:,idx(7));feature8 =
AllData(:,idx(8));feature9 = AllData(:,idx(9));feature10 =
AllData(:,idx(10)); FeaturesSelected =
horzcat(feature1,feature2,feature3,feature4,feature5,fs_Y);colNames =
{&#39;1&#39;,&#39;2&#39;,&#39;3&#39;,&#39;4&#39;,&#39;5&#39;,&#39;Label&#39;
};FeaturesSelected_Table =
array2table(FeaturesSelected,&#39;VariableNames&#39;,colNames);%%ratio21 =
feature2./feature1;ratio31 = feature3./feature1;ratio32 =
feature3./feature2;ratio = horzcat(ratio21,ratio31,fs_Y); colNames = {&#39;ratio
450nm/401nm&#39;,&#39;ratio 480nm/401nm&#39;,&#39;Label&#39;};ratio_Table =
array2table(ratio,&#39;VariableNames&#39;,colNames);
```

**Code for machine learning:**

```
database =
readtable(&#39;E:\Ashwini\Matlab\Results\2020_01_28\20200128_1139Floro_Input_NC.x
lsx&#39;,&#39;Sheet&#39;,&#39;FeatureMatrix&#39;); %FullSet_0 FFT_0 BP_Coeff
[r,c1] =
size(database);shuffledRow = randperm(r);database = database(shuffledRow,
:);disp(c1)columLength = size(database);X= database(:, 1:columLength(2)-1);Y
= database(:, columLength(2));X = table2array(X);Y = table2array(Y);% Create
```

```
a cvpartition object that defined the folds c = cvpartition(Y,&#39;holdout&#39;,0.2);
class1 = &#39;Normal&#39;; class2 = &#39;Cancer&#39;;% % Create a training set and test
setX_Train = X(training(c,1),:);Y_Train = Y(training(c,1));X_Test =
X(test(c,1),:);Y_Test = Y(test(c,1)); yourFolder =
&#39;E:\Ashwini\Matlab\Results\&#39;;newSubFolder =
[yourFolder,datestr(now,&#39;yyyy_mm_dd&#39;)];if ~exist(newSubFolder,
&#39;dir&#39;)
mkdir(newSubFolder);endfilenameReport =
[newSubFolder,&#39;\&#39;,datestr(now,&#39;yyyymmdd_HHMM&#39;),&#39;NC_Flor
oscenceReport_Final.xl
sx&#39;]; %% ANN_Algo = &#39;trainscg&#39;;net = feedforwardnet([100
10],ANN_Algo);netnet.trainFcn = ANN_Algo;net.divideParam.trainRatio =
100/100;net.divideParam.valRatio = 0/100;net.divideParam.testRatio =
0/100;net.trainParam.epochs =1000;net.trainParam.goal = 1e-
8;net.trainParam.lr = 1e-8;net.trainParam.max_fail =
100;net.trainParam.min_grad=1e-10; net.layers{1}.transferFcn = &#39;tansig&#39;;
net.layers{2}.transferFcn = &#39;tansig&#39;;net.plotFcns =
{&#39;plotperform&#39;,&#39;plottrainstate&#39;,&#39;plotconfusion&#39;,&#39;plotroc
&#39;,&#39;plotregression&#39;};[
net,tr] = train(net,X_Train&#39;,Y_Train&#39;); % TODO - TASK: Predict
responsescoreTest = net(X_Test&#39;);yT = (Y_Test);yP =
((scoreTest&#39;));figureplotconfusion(yT&#39;,yP&#39;)title([&#39;Confusion Matrix of
Test
Data using :&#39;,ANN_Algo])fh = gcf;% access the figure handle for the confusion
matrix plotah = fh.Children(2); % access the corresponding axes
```

```
handleah.XTickLabel{1} = class1;ah.XTickLabel{2} = class2 ;ah.YTickLabel{1} =
class1;ah.YTickLabel{2} = class2;ah.XLabel.String = &#39;Desired&#39;; % change the
axes labelsah.YLabel.String = &#39;Predicted&#39;; % figure[XC,YC,TC,AUCC] =
perfcurve(Y_Test&#39;,scoreTest,&#39;1&#39;);plot(XC,YC);xlabel(&#39;False positive
rate&#39;)ylabel(&#39;True positive rate&#39;)title([&#39;ROC for Classification by :
&#39;,ANN_Algo]);legend(&#39;Cancer&#39;)disp(AUCC);disp(TC); ypredicted =
cell(1,length(scoreTest));for i= 1 : length(scoreTest) if (scoreTest(i) &gt;=
0.5) ypredicted{i} = 1; else ypredicted{i} = 0; endend
s1= cell2mat(ypredicted)&#39;;s2 = Y_Test;s3 = s1==s2;s3 = s3&#39;;Match
=cell(1,length(s3));for i= 1 : length(s3) if (s3(i)==1)
Match{i} = &#39;Yes&#39;; else Match{i} = &#39;No&#39;; endend
fprintf(&#39;Prediction Accuracy(perc): %f\n&#39;, mean(double(s1 == s2)) *
100);fprintf(&#39;Area Under the Curve: %f\n&#39;,AUCC); header =
{&#39;ScoreValue&#39;,&#39;Predicted&#39;,&#39;Desired&#39;,&#39;Match(Y/N)&#3
9;};sheetname = ANN_Algo;
xlswrite(filenameReport,header,sheetname);
xlswrite(filenameReport,scoreTest&#39;,sheetname,sprintf(&#39;A2&#39;));xlswrite(filena
me
Report,s1,sheetname,sprintf(&#39;B2&#39;));xlswrite(filenameReport,s2,sheetname,sprin
tf(&#39;C2&#39;));xlswrite(filenameReport,Match&#39;,sheetname,sprintf(&#39;D2&#39;
));tp=0;tn=0;f
p=0;fn=0;s1= cell2mat(ypredicted)&#39;;for i=1:length(Y_Test) if Y_Test(i)&gt;0
if Y_Test(i)==s1(i) tp=tp+1; else fn=fn+1;
end end if Y_Test(i)&lt;=0 if Y_Test(i)==s1(i) tn=tn+1;
else fp=fp+1; end endend cm=[tn fp; fn
```

tp];disp(cm)acc=((tp+tn)/(tp+fn+fp+tn))*100;sens=(tp/(tp+fn))*100;spec=(tn/(t
n+fp))*100;prec=(tp/(tp+fp))*100;NPV=(tn/(tn+fn))*100;FNR=(fn/(tp+fn))*100;FP
R=(fp/(tn+fp))*100;FDR=(fp/(tp+fp))*100;FOR=(fn/(tn+fn))*100;fm=((2*prec*sens
)/(prec+sens));missRate = ((fp+fn)/(tp+fn+fp+tn))*100;disp(['Accuracy:
',num2str(acc),'%'])disp(['Misclassification Rate:
',num2str(missRate),'%'])disp(['Sensitivity:
',num2str(sens),'%'])disp(['Specificity:
',num2str(spec),'%'])disp(['Precision:
',num2str(prec),'%'])disp(['Negative
Predictive Value: ',num2str(NPV),'%'])disp(['False negative rate:
',num2str(FNR),'%'])disp(['False positive rate:
',num2str(FPR),'%'])disp(['False discovery rate:
',num2str(FDR),'%'])disp(['False Omission rate:
',num2str(FOR),'%'])disp(['F
Score: ',num2str(fm)]) %%% weighted average of the true positive rate
(recall) and precision %% SVM SVMKernalFunction = 'rbf';MdlSVM =
fitcsvm(X_Train,Y_Train,'ClassNames',[0
1],'KernelFunction',SVMKernalFunction,'Standardize',true,
'OptimizeHyperparameters','auto',...
'HyperparameterOptimizationOptions',struct('AcquisitionFunctionName&#
39;,...
'expected-improvement-plus','ShowPlots',false)); [Predicted_Output,
scores] =
predict(MdlSVM,X_Test);fprintf('Prediction Accuracy(perc): %f\n',
mean(double(Predicted_Output == Y_Test)) * 100);C =

confusionmat(Y_Test,Predicted_Output);figurelabel={class1,class2};confusionch

art(C,label);xlabel('Desired')ylabel('Predicted')title(['Confusion

Matrix of

Test Data using SVM Model function:',SVMKernalFunction]) % figure [X,Y,~,AUC]

= perfcurve(Y_Test,(scores(:,2)),1);plot(X,Y,'LineWidth',2)xlabel('False

positive rate')ylabel('True positive rate')title('ROC for Classification

')

fprintf('AUC Value :%f\n',AUC)%%% Calculate the loss errTrain and

errTesterrTrain = resubLoss(MdlSVM);errTest = loss(MdlSVM,X_Test,Y_Test);%%%

Display the resultsdisp(['Training Error: ',num2str(errTrain)])disp(['Test

Error: ',num2str(errTest)]) scoreTest = scores(:,2);ypredicted =

cell(1,length(scoreTest));for i= 1 : length(scoreTest) if (scoreTest(i) >

0) ypredicted{i} = 1; else ypredicted{i} = 0; endend %s1=

cell2mat(ypredicted)';s2 = Y_Test;s3 = Predicted_Output==s2;s3 = s3'; Match

=cell(1,length(s3));for i= 1 : length(s3) if (s3(i)==1)

Match{i} = 'Yes'; else Match{i} = 'No'; endendheader =

{'ScoreValue','Predicted','Desired','Match(Y/N)&#3

9;};sheetname =

['SVM_',SVMKernalFunction]; xlswrite(filenameReport,header,sheetname);

xlswrite(filenameReport,scores(:,2),sheetname,sprintf('A2'));xlswrite(filenam

eReport,Predicted_Output,sheetname,sprintf('B2'));xlswrite(filenameReport,s2,

sheetname,sprintf('C2'));xlswrite(filenameReport,Match',sheetname,sprintf(

'D2

')); tp=0;tn=0;fp=0;fn=0;s1= Predicted_Output;for i=1:length(Y_Test) if

Y_Test(i)>0 if Y_Test(i)==s1(i) tp=tp+1; else

```
fn=fn+1; end end if Y_Test(i)&lt;=0 if Y_Test(i)==s1(i)
tn=tn+1; else fp=fp+1; end endend cm=[tn fp; fn
tp];disp(cm)acc=((tp+tn)/(tp+fn+fp+tn))*100;sens=(tp/(tp+fn))*100;spec=(tn/(t
n+fp))*100;prec=(tp/(tp+fp))*100;NPV=(tn/(tn+fn))*100;FNR=(fn/(tp+fn))*100;FP
R=(fp/(tn+fp))*100;FDR=(fp/(tp+fp))*100;FOR=(fn/(tn+fn))*100;fm=((2*prec*sens
)/(prec+sens));missRate = ((fp+fn)/(tp+fn+fp+tn))*100;disp([&#39;Accuracy:
&#39;,num2str(acc),&#39;%&#39;])disp([&#39;Misclassification Rate:
&#39;,num2str(missRate),&#39;%&#39;])disp([&#39;Sensitivity:
&#39;,num2str(sens),&#39;%&#39;])disp([&#39;Specificity:
&#39;,num2str(spec),&#39;%&#39;])disp([&#39;Precision:
&#39;,num2str(prec),&#39;%&#39;])disp([&#39;Negative
Predictive Value: &#39;,num2str(NPV),&#39;%&#39;])disp([&#39;False negative rate:
&#39;,num2str(FNR),&#39;%&#39;])disp([&#39;False positive rate:
&#39;,num2str(FPR),&#39;%&#39;])disp([&#39;False discovery rate:
&#39;,num2str(FDR),&#39;%&#39;])disp([&#39;False Omission rate:
&#39;,num2str(FOR),&#39;%&#39;])disp([&#39;F
Score: &#39;,num2str(fm)]) %%% weighted average of the true positive rate
(recall) and precision %% Decision Tree MdlDT =
fitctree(X_Train,Y_Train,&#39;ClassNames&#39;,[0
1],&#39;OptimizeHyperparameters&#39;,&#39;auto&#39;,...
&#39;HyperparameterOptimizationOptions&#39;,struct(&#39;AcquisitionFunctionName&#
39;,...
&#39;expected-improvement-plus&#39;,&#39;ShowPlots&#39;,false));[Predicted_Output,
scores] =
predict(MdlDT, X_Test);fprintf(&#39;Prediction Accuracy(perc): %f\n&#39;
```

```
, mean(double(Predicted_Output == Y_Test)) * 100);C =
confusionmat(Y_Test,Predicted_Output);figurelabel={class1,class2};confusionch
art(C,label);title(&#39;Confusion Matrix of Test Data using Decision
Tree&#39;)xlabel(&#39;Desired&#39;)ylabel(&#39;Predicted&#39;) figure[X,Y,~,AUC] =
perfcurve(Y_Test,(scores(:,2)),1);plot(X,Y,&#39;LineWidth&#39;,2)xlabel(&#39;False
positive rate&#39;)ylabel(&#39;True positive rate&#39;)title(&#39;ROC for Classification
&#39;)fprintf(&#39;AUC Value :%f\n&#39;,AUC)%%% Calculate the loss errTrain and
errTesterrTrain = resubLoss(MdlDT);errTest = loss(MdlDT,X_Test,Y_Test);%%%
Display the resultsdisp([&#39;Training Error: &#39;,num2str(errTrain)])disp([&#39;Test
Error: &#39;,num2str(errTest)]) view(MdlDT,&#39;Mode&#39;,&#39;graph&#39;)
scoreTest =
scores(:,2);ypredicted = cell(1,length(scoreTest));for i= 1 :
length(scoreTest) if (scoreTest(i) &gt; 0.5) ypredicted{i} = 1;
else ypredicted{i} = 0; endend s1= Predicted_Output;s2 = Y_Test;s3
= s1==s2;s3 = s3&#39;; Match =cell(1,length(s3));for i= 1 : length(s3) if
(s3(i)==1) Match{i} = &#39;Yes&#39;; else Match{i} = &#39;No&#39;; endend
header =
{&#39;ScoreValue&#39;,&#39;Predicted&#39;,&#39;Desired&#39;,&#39;Match(Y/N)&#3
9;};sheetname =
&#39;DecisionTree&#39;; xlswrite(filenameReport,header,sheetname);
xlswrite(filenameReport,scores(:,1),sheetname,sprintf(&#39;A2&#39;));xlswrite(filenam
eReport,Predicted_Output,sheetname,sprintf(&#39;B2&#39;));xlswrite(filenameReport,s2,
sheetname,sprintf(&#39;C2&#39;));xlswrite(filenameReport,Match&#39;,sheetname,sprintf(
&#39;D2
&#39;)); tp=0;tn=0;fp=0;fn=0;s1= cell2mat(ypredicted)&#39;;for i=1:length(Y_Test)
```

```
if Y_Test(i)&gt;0 if Y_Test(i)==s1(i) tp=tp+1; else
fn=fn+1; end end if Y_Test(i)&lt;=0 if Y_Test(i)==s1(i)
tn=tn+1; else fp=fp+1; end endend cm=[tn fp; fn
tp];disp(cm)acc=((tp+tn)/(tp+fn+fp+tn))*100;sens=(tp/(tp+fn))*100;spec=(tn/(t
n+fp))*100;prec=(tp/(tp+fp))*100;NPV=(tn/(tn+fn))*100;FNR=(fn/(tp+fn))*100;FP
R=(fp/(tn+fp))*100;FDR=(fp/(tp+fp))*100;FOR=(fn/(tn+fn))*100;fm=((2*prec*sens
)/(prec+sens));missRate = ((fp+fn)/(tp+fn+fp+tn))*100;disp([&#39;Accuracy:
&#39;,num2str(acc),&#39;%&#39;])disp([&#39;Misclassification Rate:
&#39;,num2str(missRate),&#39;%&#39;])disp([&#39;Sensitivity:
&#39;,num2str(sens),&#39;%&#39;])disp([&#39;Specificity:
&#39;,num2str(spec),&#39;%&#39;])disp([&#39;Precision:
&#39;,num2str(prec),&#39;%&#39;])disp([&#39;Negative
Predictive Value: &#39;,num2str(NPV),&#39;%&#39;])disp([&#39;False negative rate:
&#39;,num2str(FNR),&#39;%&#39;])disp([&#39;False positive rate:
&#39;,num2str(FPR),&#39;%&#39;])disp([&#39;False discovery rate:
&#39;,num2str(FDR),&#39;%&#39;])disp([&#39;False Omission rate:
&#39;,num2str(FOR),&#39;%&#39;])disp([&#39;F

Score: &#39;,num2str(fm)]) %%% weighted average of the true positive rate
(recall) and precision %% naive Bayes MdlNB =
fitcnb(X_Train,Y_Train,&#39;ClassNames&#39;,[0
1],&#39;OptimizeHyperparameters&#39;,&#39;auto&#39;,...
&#39;HyperparameterOptimizationOptions&#39;,struct(&#39;AcquisitionFunctionName&#
39;,...
```

```
&#39;expected-improvement-plus&#39;,&#39;ShowPlots&#39;,false));[Predicted_Output,
scores] =
predict(MdlNB, X_Test);fprintf(&#39;Prediction Accuracy(perc): %f\n&#39;,
mean(double(Predicted_Output == Y_Test)) * 100);C =
confusionmat(Y_Test,Predicted_Output);figurelabel={class1,class2};confusionch
art(C,label);title(&#39;Confusion Matrix of Test Data using Naive
Bayes&#39;)xlabel(&#39;Desired&#39;)ylabel(&#39;Predicted&#39;) figure[X,Y,~,AUC]
=
perfcurve(Y_Test,(scores(:,2)),1);plot(X,Y,&#39;LineWidth&#39;,2)xlabel(&#39;False
positive rate&#39;)ylabel(&#39;True positive rate&#39;)title(&#39;ROC for Classification
&#39;)fprintf(&#39;AUC Value :%f\n&#39;,AUC)%%% Calculate the loss errTrain and
errTesterrTrain = resubLoss(MdlNB);errTest = loss(MdlNB,X_Test,Y_Test);%%%
Display the resultsdisp([&#39;Training Error: &#39;,num2str(errTrain)])disp([&#39;Test
Error: &#39;,num2str(errTest)]) scoreTest = scores(:,1);ypredicted =
cell(1,length(scoreTest));for i= 1 : length(scoreTest) if (scoreTest(i) &gt;
0.1) ypredicted{i} = 1; else ypredicted{i} = 0; endend
s1= cell2mat(ypredicted)&#39;;s2 = Y_Test;s3 = s1==s2;s3 = s3&#39;; Match
=cell(1,length(s3));for i= 1 : length(s3) if (s3(i)==1)
Match{i} = &#39;Yes&#39;; else Match{i} = &#39;No&#39;; endend header =
{&#39;ScoreValue&#39;,&#39;Predicted&#39;,&#39;Desired&#39;,&#39;Match(Y/N)&#3
9;};sheetname = &#39;Naive Bayes&#39;;
xlswrite(filenameReport,header,sheetname);
xlswrite(filenameReport,scores(:,1),sheetname,sprintf(&#39;A2&#39;));xlswrite(filenam
eReport,Predicted_Output,sheetname,sprintf(&#39;B2&#39;));xlswrite(filenameReport,s2,
```

```
sheetname,sprintf(&#39;C2&#39;));xlswrite(filenameReport,Match&#39;,sheetname,sprintf(
&#39;D2
&#39;));tp=0;tn=0;fp=0;fn=0; s1= Predicted_Output;for i=1:length(Y_Test) if
Y_Test(i)&gt;0 if Y_Test(i)==s1(i) tp=tp+1; else
fn=fn+1; end end if Y_Test(i)&lt;=0 if Y_Test(i)==s1(i)
tn=tn+1; else fp=fp+1; end endend cm=[tn fp; fn
tp];disp(cm)acc=((tp+tn)/(tp+fn+fp+tn))*100;sens=(tp/(tp+fn))*100;spec=(tn/(t
n+fp))*100;prec=(tp/(tp+fp))*100;NPV=(tn/(tn+fn))*100;FNR=(fn/(tp+fn))*100;FP
R=(fp/(tn+fp))*100;FDR=(fp/(tp+fp))*100;FOR=(fn/(tn+fn))*100;fm=((2*prec*sens
)/(prec+sens));missRate = ((fp+fn)/(tp+fn+fp+tn))*100;disp([&#39;Accuracy:
&#39;,num2str(acc),&#39;%&#39;])disp([&#39;Misclassification Rate:
&#39;,num2str(missRate),&#39;%&#39;])disp([&#39;Sensitivity:
&#39;,num2str(sens),&#39;%&#39;])disp([&#39;Specificity:
&#39;,num2str(spec),&#39;%&#39;])disp([&#39;Precision:
&#39;,num2str(prec),&#39;%&#39;])disp([&#39;Negative
Predictive Value: &#39;,num2str(NPV),&#39;%&#39;])disp([&#39;False negative rate:
&#39;,num2str(FNR),&#39;%&#39;])disp([&#39;False positive rate:
&#39;,num2str(FPR),&#39;%&#39;])disp([&#39;False discovery rate:
&#39;,num2str(FDR),&#39;%&#39;])disp([&#39;False Omission rate:
&#39;,num2str(FOR),&#39;%&#39;])disp([&#39;F
Score: &#39;,num2str(fm)]) %%% weighted average of the true positive rate
(recall) and precision%%%%%%Linear - logistic Regression MdlLinear =
fitclinear(X_Train&#39;,Y_Train,&#39;ClassNames&#39;,[0
1],&#39;ObservationsIn&#39;,&#39;columns&#39;,&#39;Solver&#39;,&#39;sparsa&#39;,.
..
```

```
&#39;OptimizeHyperparameters&#39;,&#39;auto&#39;,&#39;HyperparameterOptimization
Options&#39;,...
struct(&#39;AcquisitionFunctionName&#39;,&#39;expected-improvement-
plus&#39;,&#39;ShowPlots&#39;,false));[Predicted_Output, scores] = predict(MdlLinear,
X_Test);fprintf(&#39;Prediction Accuracy(perc): %f\n&#39;,
mean(double(Predicted_Output == Y_Test)) * 100); C =
confusionmat(Y_Test,Predicted_Output);figurelabel={class1,class2};confusionch
art(C,label);title(&#39;Confusion Matrix of Test Data using Logistic
Regresssion&#39;)xlabel(&#39;Desired&#39;)ylabel(&#39;Predicted&#39;)
figure[X,Y,T,AUC] =
perfcurve(Y_Test,(scores(:,2)),1);plot(X,Y,&#39;LineWidth&#39;,2)xlabel(&#39;False
positive rate&#39;)ylabel(&#39;True positive rate&#39;)title(&#39;ROC for Classification
&#39;)fprintf(&#39;AUC Value :%f\n&#39;,AUC)%%% Calculate the loss errTrain and
errTest%errTrain = resubLoss(MdlLinear);errTest =
loss(MdlLinear,X_Test,Y_Test);%%% Display the results%disp([&#39;Training Error:
&#39;,num2str(errTrain)])disp([&#39;Test Error: &#39;,num2str(errTest)]) scoreTest =
scores(:,2);ypredicted = cell(1,length(scoreTest));for i= 1 :
length(scoreTest) if (scoreTest(i) &gt; 0.5) ypredicted{i} = 1;
else ypredicted{i} = 0; endend s1= cell2mat(ypredicted)&#39;;s2 =
Y_Test;s3 = s1==s2;s3 = s3&#39;; Match =cell(1,length(s3));for i= 1 : length(s3)
if (s3(i)==1) Match{i} = &#39;Yes&#39;; else Match{i} = &#39;No&#39;;
endend header =
{&#39;Desired&#39;,&#39;ScoreValue&#39;,&#39;Predicted&#39;,&#39;Match(Y/N)&#3
9;};sheetname =
&#39;Logistic Regression&#39;;
```

```
xlswrite(filenameReport,header,sheetname);xlswrite(filenameReport,s2,sheetnam
e,sprintf(&#39;A2&#39;));xlswrite(filenameReport,scores(:,2),sheetname,sprintf(&#39;B2&
#39;))
;xlswrite(filenameReport,Predicted_Output,sheetname,sprintf(&#39;C2&#39;));xlswrite(f
ilenameReport,Match&#39;,sheetname,sprintf(&#39;D2&#39;)); tp=0;tn=0;fp=0;fn=0; s1=
cell2mat(ypredicted)&#39;;for i=1:length(Y_Test) if Y_Test(i)&gt;0 if
Y_Test(i)==s1(i) tp=tp+1; else fn=fn+1;
end end if Y_Test(i)&lt;=0 if Y_Test(i)==s1(i) tn=tn+1;
else fp=fp+1; end endend cm=[tn fp; fn
tp];disp(cm)acc=((tp+tn)/(tp+fn+fp+tn))*100;sens=(tp/(tp+fn))*100;spec=(tn/(t
n+fp))*100;prec=(tp/(tp+fp))*100;NPV=(tn/(tn+fn))*100;FNR=(fn/(tp+fn))*100;FP
R=(fp/(tn+fp))*100;FDR=(fp/(tp+fp))*100;FOR=(fn/(tn+fn))*100;fm=((2*prec*sens
)/(prec+sens));missRate = ((fp+fn)/(tp+fn+fp+tn))*100;disp([&#39;Accuracy:
&#39;,num2str(acc),&#39;%&#39;])disp([&#39;Misclassification Rate:
&#39;,num2str(missRate),&#39;%&#39;])disp([&#39;Sensitivity:
&#39;,num2str(sens),&#39;%&#39;])disp([&#39;Specificity:
&#39;,num2str(spec),&#39;%&#39;])disp([&#39;Precision:
&#39;,num2str(prec),&#39;%&#39;])disp([&#39;Negative
Predictive Value: &#39;,num2str(NPV),&#39;%&#39;])disp([&#39;False negative rate:
&#39;,num2str(FNR),&#39;%&#39;])disp([&#39;False positive rate:
&#39;,num2str(FPR),&#39;%&#39;])disp([&#39;False discovery rate:
&#39;,num2str(FDR),&#39;%&#39;])disp([&#39;False Omission rate:
&#39;,num2str(FOR),&#39;%&#39;])disp([&#39;F
Score: &#39;,num2str(fm)]) %%% weighted average of the true positive rate
(recall) and precision%% Cross validation value indices =
```

crossvalind('Kfold',,Y,5);cp = classperf(Y);for i = 1:5test = (indices == i);

train = ~test;class =

classify(X(test,:),X(train,:),Y(train,:),'linear');classperf(cp,class,test)en

dcp.ErrorRate

## REFERENCES


[1] Peng, H., Long, F. and Ding, C. (2005). Feature selection based on mutual information criteria of max-dependency, max-relevance, and min-redundancy. *IEEE Transactions on pattern analysis and machine intelligence*, *27*(8), 1226-1238.

[2] Kahraman, C. ed. (2012). Computational intelligence systems in industrial engineering: With recent theory and applications. Springer Science & Business Media.

[3] Bonaccorso, G. (2018). Mastering Machine Learning Algorithms: Expert techniques to implement popular machine learning algorithms and fine-tune your models. Packt Publishing Ltd.

[4] Møller, M.F. (1993). A scaled conjugate gradient algorithm for fast supervised learning. Neural networks, 6(4),.525-533.

[5] Platt, J. (1999). “Probabilistic outputs for support vector machines and comparisons to regularized likelihood methods.” Advances in Large Margin Classifiers. MIT Press, 61–74.

[6] Schölkopf, B., Smola, A.J. and Bach, F. (2002). Learning with kernels: support vector machines, regularization, optimization, and beyond. MIT press.

[7] Bonaccorso, G. (2018). Mastering Machine Learning Algorithms: Expert techniques to implement popular machine learning algorithms and fine-tune your models. Packt Publishing Ltd.

[8] Platt, J. (1999). “Probabilistic outputs for support vector machines and comparisons to regularized likelihood methods.” Advances in Large Margin Classifiers. MIT Press, 61–74.

[9] Rish, I., 2001, August. An empirical study of the naive Bayes classifier. In *IJCAI 2001 workshop on empirical methods in artificial intelligence* 3(22), 41-46.

[10] Subasi, A. and Yaman, E. (2019). EMG Signal Classification Using Discrete Wavelet Transform and Rotation Forest. In International Conference on Medical and Biological Engineering.73, 29-35.

[11] Ozdemir, S. (2016). *Principles of Data Science*. Packt Publishing Ltd.

[12] Sammut, C. and Webb, G.I. (2017). *Encyclopedia of machine learning and data mining*. Springer Publishing Company, Incorporated.

[13] Hajian-Tilaki, K. (2013). Receiver operating characteristic (ROC) curve analysis for medical diagnostic test evaluation. *Caspian journal of internal medicine*, *4*(2), 627.

[14] Chicco, D and Jurman, G (2020). The advantages of the Matthews correlation coefficient (MCC) over F1 score and accuracy in binary classification evaluation. *BMC genomics,* 21(1), 6.
